# Supramolecular Aggregation-Induced Emission Photosensitizer Encapsulated by Cucurbit[8]uril Nanocavity Boosts Hypoxia-Activated Tumor Therapy

**DOI:** 10.1021/acsanm.5c03879

**Published:** 2025-10-18

**Authors:** Wen Liu, Zeyu Zhang, Qing Huang, Rishi Pai, Lucas Liu, Zufeng Ding, Xing Wang, Zhicheng Jin

**Affiliations:** a Department of Chemistry, 1373 Georgia State University, Atlanta, Georgia 30303, United States; b Department of Biology, 1373 Georgia State University, Atlanta, Georgia 30303, United States

**Keywords:** hypoxia, aggregation induced emission (AIE), supramolecular photosensitizer, self-assembly, photodynamic therapy

## Abstract

The limited efficacy of chemotherapy induced by the hypoxic tumor microenvironment remains a major obstacle in clinical oncology. To address this, we designed and synthesized a series of aggregation-induced emission photosensitizers with donor−π–acceptor structures, exemplified by SC-3. Upon assembly with cucurbit[8]­uril (CB[8]), the nanosupramolecular photosensitizer 2SC-3/CB[8] significantly enhanced singlet oxygen (^1^O_2_) generation and exacerbated cellular hypoxia. When coadministered with the hypoxia-activated chemotherapeutic tirapazamine (TPZ), we found that 2SC-3/CB[8] further amplified TPZ’s antitumor efficacy in MDA-MB-231 breast cancer cells. Experimental studies and density functional theory calculations confirmed that SC-3 exhibits potent photodynamic therapy activity with high mitochondrial specificity. In addition, we found that CB[8]-induced depletion of intracellular spermine further contributed to the death of the tumor cells. Together, these findings highlight the potential of combining supramolecular photosensitizers with hypoxia-activated chemotherapy as a promising and synergistic strategy for the treatment of solid tumors.

## Graphical abstract

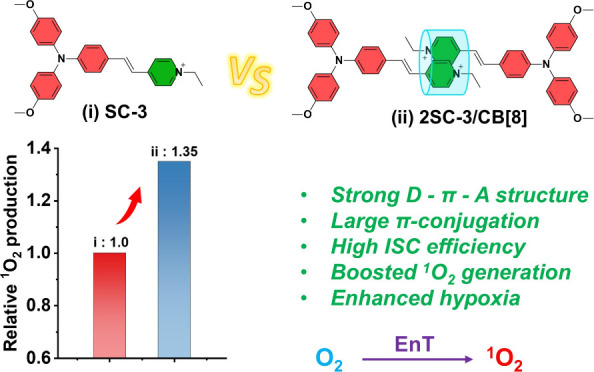

## 1 Introduction

Breast cancer is a prevalent malignancy in women.^1–4^ Rapid growth, high metabolic demand, and inadequate neovascularization create a hypoxic microenvironment. This hypoxia drives invasion, metastasis, and therapy resistance.^5–9^ Consequently, targeting the hypoxic microenvironment has emerged as a pivotal therapeutic strategy. For example, tirapazamine (TPZ) is a hypoxia-activated antitumor agent that induces DNA damage via cytotoxic free radicals.^10–13^ Currently, its efficacy is constrained by limited intratumoral biodistribution, and strategies that improve tumor penetration and intensify hypoxia may enhance the clinical performance of hypoxia-activated therapies.^14,15^ Recent studies focus on combining TPZ with other drugs, developing delivery systems, and designing smart carriers to enhance its specificity and efficacy.^16–18^

Tumor hypoxia can be intensified by reactive oxygen species (ROS) generation, which consume local oxygen and reduce cellular oxygen levels. Photodynamic therapy (PDT) leverages this principle by using photosensitizers, light, and oxygen to produce cytotoxic ROS.^19–22^ Due to its noninvasiveness, precision, and mild side effects, PDT is widely used in oncology, dermatology, and antimicrobial therapy.^23–25^ Conventional PDT agents, such as porphyrin- and phthalocyanine-based photosensitizers, though efficient as monomers, suffer from aggregation-caused quenching in aqueous environments, limiting ROS generation and PDT efficacy.^26–30^ In contrast, aggregation-induced emission (AIE) photosensitizers enhance ROS production by restricting intramolecular motions in the aggregated state and promoting intersystem crossing (ISC) via donor–acceptor (D-A) architectures or heavy atoms.^31–36^ By simultaneously generating ROS and consuming local oxygen, AIE photosensitizers both induce tumor cell death and exacerbate hypoxia, thereby enhancing the efficacy of hypoxia-activated therapies like TPZ when delivered through supramolecular or nanocarriers. Supramolecular carriers, such as cucurbituril, further enhance the antitumor performance of AIE photosensitizers. The rigid cavity and strong host–guest recognition of cucurbit[8]­uril (CB[8]) improve photosensitizer stability and boost ROS generation.^37,38^ Mechanistic studies show that when CB[8] forms stable complexes with cationic AIE photosensitizers via electrostatic and hydrophobic interactions, it restricts rotational and vibrational motions, suppressing nonradiative decay and promoting ISC from singlet to triplet states.^39–42^ Furthermore, encapsulation of multiple guest photosensitizers can induce intermolecular charge-transfer complexes, further facilitating ISC, increasing ROS production, and enhancing PDT efficacy.

In this work, we designed and synthesized four AIE photosensitizers (SC-*n*, *n* = 1–4) with donor−π–acceptor (D-π-A) structures via donor modulation. Both experimental results and density functional theory (DFT) calculations confirmed that SC-3 exhibits potent tumor-targeted activity with mitochondrial specificity. The nanosupramolecular photosensitizer 2SC-3/CB[8], formed via self-assembly of SC-3 and CB[8], enhances ^1^O_2_ generation by 35% compared to SC-3 alone. We also found that the abundant ^1^O_2_ ROS exacerbates oxidative stress and activates the cytochrome c/caspase9/caspase3 apoptotic pathway. As shown in Figure 1, when combined with the hypoxia-activated chemotherapeutic TPZ in MDA-MB-231 breast cancer cells, PDT based on 2SC-3/CB[8] intensifies tumor hypoxia and enhances TPZ’s antitumor efficacy. Additionally, CB[8]-mediated spermine depletion also contributes to tumor cell death. Together, our nanosupramolecular photosensitizer 2SC-3/CB[8] combined with TPZ provide a promising approach for the treatment of solid tumors, addressing challenges in tumor targeting, ROS generation, as well as hypoxia-activated therapy.

**Figure 1:**
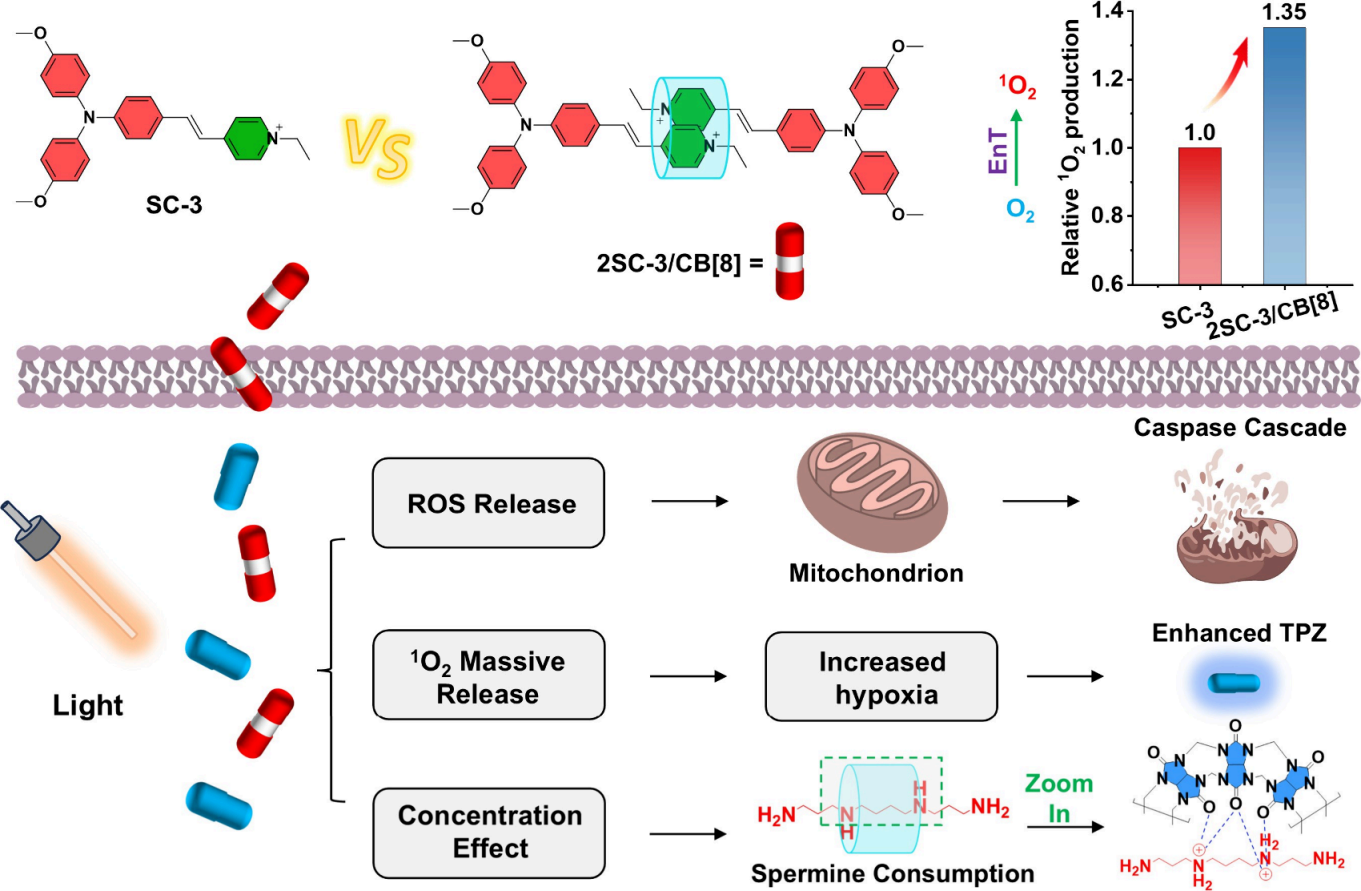
Schematic illustration of the design of supramolecular photosensitizer (2SC-3/CB[8], red capsule) to enhance ^1^O_2_ generation and its synergistic mechanism with hypoxia-activated chemotherapeutic tirapazamine (TPZ, blue capsule) for improved tumor therapy.

## 2 Experimental Methods

### 2.1 Materials and Characterization

All reagents employed for the synthesis of the target compounds in this study were obtained from Sigma-Aldrich and Thermo Scientific, and were used directly without further purification. Chemicals used for the photophysical characterization of the target compounds were obtained from Sigma-Aldrich. The human triple-negative breast cancer MDA-MB-231 cells, human umbilical vein endothelial cells (HUVECs), phosphate buffer solution (PBS), trypsin, fetal bovine serum (FBS), and Dulbecco’s Modified Eagle Medium (DMEM) incomplete medium for cell culture, were purchased from Thermo Scientific. The assay kits used for the detection of specific substances in MDA-MB-231 cells were obtained from Thermo Scientific. Ready-to-use reagents including 3-(4,5-dimethylthiazol-2-yl)-2,5-diphenyltetrazolium bromide (MTT), 2’,7’-dichlorodihydrofluorescein (DCFH), dihydrorhodamine 123 (DHR123), hydroxyphenyl fluorescein (HPF), singlet oxygen sensor green (SOSG), Mito-Tracker Green probe, antibodies (rabbit anticytochrome c/mouse anti-caspase9/mouse anti-caspase3) were purchased from Thermo Scientific. ^1^H and ^13^C nuclear magnetic resonance (NMR) spectra of intermediate and target compounds were recorded on Bruker AV II-600 MHz NMR spectrometer and analyzed via MestReNova to confirm the structures. Chemical shifts (δ) were referenced to tetramethylsilane (TMS, δ = 0 ppm) and recorded in DMSO-*d*_6_ or DMSO-*d*_6_/D_2_O as indicated. Mass spectrometry data were acquired via Waters Xevo-X2_GS (ESI-QTof). Ultraviolet–visible (UV–vis) absorption spectra were measured using an Agilent Cary 100 UV–vis spectrophotometer. Fluorescence spectra were obtained from Fluoromax-4 spectrofluorometer. Confocal laser scanning microscopy images of MDA-MB-231 cells were captured with the Zeiss LSM980 system.

### 2.2 AIE Curve Determination

In the mixed solvent system of toluene (Tol) and dimethyl sulfoxide (DMSO), the spectra of aggregation-induced emission luminogens (AIEgens) at a concentration of 10 μM were recorded to obtain the AIE curves. Specifically, by varying the proportion of the poor solvent Tol within the mixture, fluorescence spectra of AIEgens under different aggregation states were also investigated. The relative changes in the maximum fluorescence intensity were quantitatively analyzed to further elucidate the aggregation behavior of the AIEgens.

### 2.3 In Vitro ROS Level Measurement

In this study, we evaluated the overall ROS generation capacity of the AIEgens, as well as their ability to produce type-I ROS, such as superoxide anion (O_2_^–•^) and hydroxyl radical (OH**·**), and type-II ROS (^1^O_2_). Specifically, aqueous solutions containing AIEgens were supplemented with 10 μM of DCFH, DHR123, HPF, or 9,10-anthracenediyl-bis­(methylene)­dimalonic acid (ABDA), followed by irradiation with white light (40 mW/cm^2^) for varying times. The fluorescence or absorption spectra of the resulting mixtures were then recorded to quantify the ROS and each free radical generation efficiencies under irradiation. The ^1^O_2_ production capacity of 2SC-3/CB[8] under light was determined following the same procedure.

### 2.4 DFT Calculation Study

The four AIEgens (SC-*n*, *n* = 1–4) involved in this study were optimized in water and their frontier molecular orbitals were analyzed via the Gaussian 16 program package.^37^ For the excited state calculation, the ground state geometry is optimized at B3LYP/6-311G­(d) level also using the Gaussian 16 program package, and the corresponding energy levels were obtained simultaneously.

### 2.5 Mitochondrial Colocalization

SC-3 and 2SC-3/CB[8] at a concentration of 10 μM were coincubated with MDA-MB-231 cells for 4 h, followed by the addition of Mito-Tracker Green (10 μM) to the culture medium. Following an additional 20 min of coincubation, mitochondrial colocalization images of MDA-MB-231 cells were obtained using confocal laser scanning microscopy. The colocalization indexes between SC-3 or 2SC-3/CB[8] and mitochondria was subsequently quantified with ImageJ.

### 2.6 Cytotoxicity Analysis

The cytotoxicity of TPZ, CB[8], SC-3, 2SC-3/CB[8], and the combination treatment (2SC-3/CB[8] + TPZ) was evaluated using the MTT assay. In the cytotoxicity experiments, TPZ was administered in its free form. Specifically, MDA-MB-231 cells were seeded into 96-well plates at a density of 6000–8000 cells per well and cultured overnight at 37 °C in a humidified 5% CO_2_ atmosphere to facilitate cell adhesion. The culture medium in each well was replaced with fresh medium containing varying concentrations of the respective compounds, with replicate wells prepared for each condition. The plates were then incubated for 24 h under atmospheres with different oxygen levels. After incubation, 10 μL of MTT solution (5 mg/mL) was added to each well and incubated for an additional 4 h. The medium was subsequently removed, and the resulting formazan crystals were dissolved in DMSO. The absorbance of each well at 490 nm was measured using a microplate reader (Thermo Scientific), and IC_50_ values were determined using nonlinear regression analysis with IBM SPSS Statistics 16. Each experiment was performed in at least three independent replicates (*n* ≥ 3). Error bars represent the standard deviation (SD), as indicated in the figure legends. For light-irradiated groups, cells were exposed to white light (40 mW/cm^2^) for 20 min at 4 h post-treatment, followed by continued incubation for 24 h before conducting the same MTT assay procedure. The toxicity of different compounds on HUVECs was also detected using this method.

## 3 Results and Discussion

### 3.1 Synthesis of AIEgens

In this study, 4-methoxy-*N*-(4-methoxyphenyl)-*N*-phenylaniline was initially converted into compound a via the Vilsmeier–Haack formylation reaction. Subsequently, demethylation of the aromatic ether was carried out using BBr_3_ at 0 °C to produce the phenolic compound b (Figure 2). The quaternary ammonium salt c, “between 4-methylpyridine and 2-bromoethane, is an electron-withdrawing moiety for the subsequent Knoevenagel condensation with various triphenylamine derivatives, yielding four types of D-π-A structured AIEgens (SC-*n*, *n* = 1–4). All intermediates and final products of SC-*n* series were fully characterized by ^1^H NMR, ^13^C NMR, and high-resolution mass spectrometry (Figures S1–S7), confirming their molecular structures.

**Figure 2:**
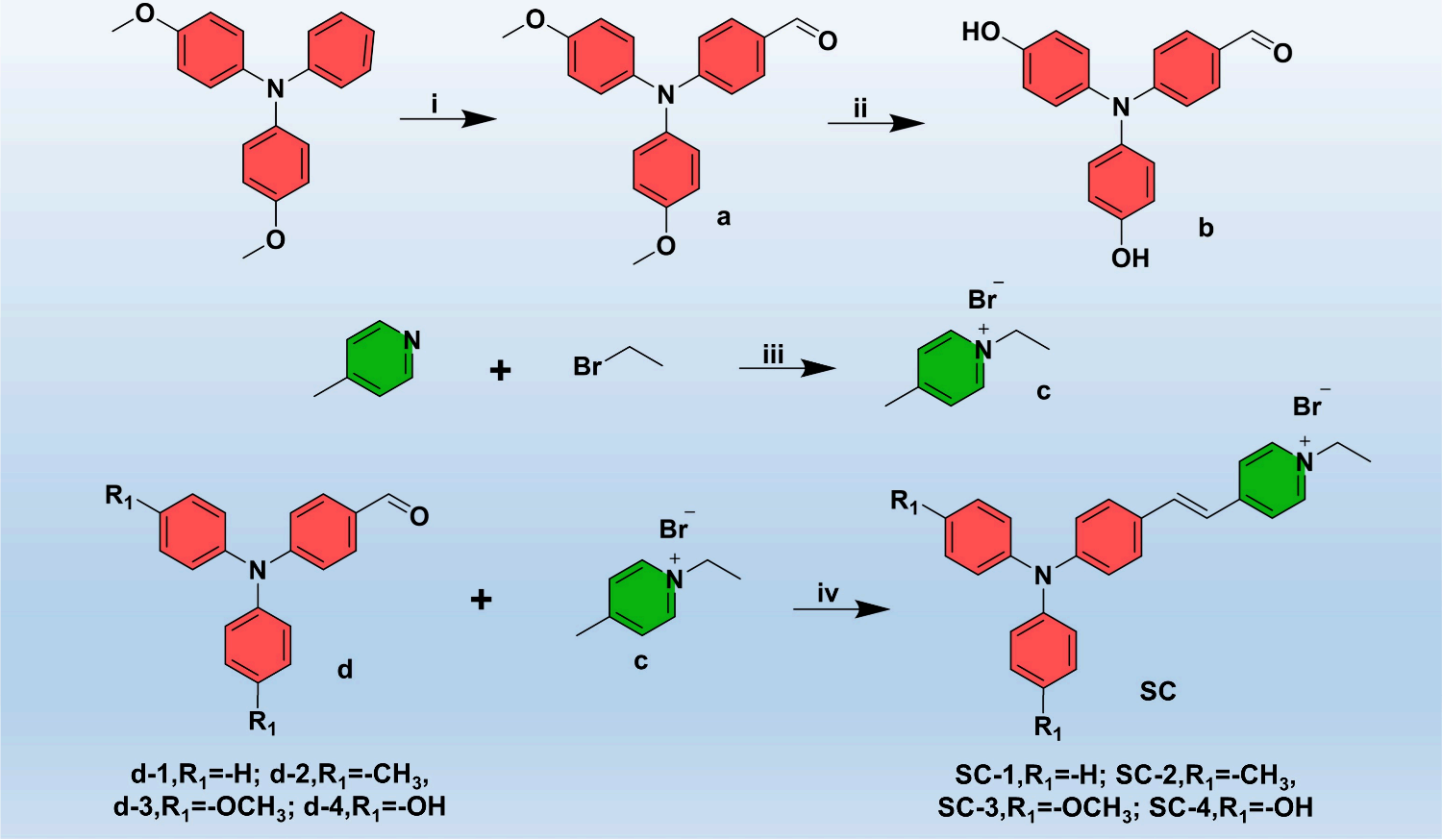
Synthetic route of four D-π-A structured AIEgens (SC-*n*, *n* = 1–4) with varying R_1_ substituents. The reaction conditions include (i) POCl_3_, *N*,*N*-dimethylformamide, 80 °C, 24 h; (ii) BBr_3_, dichloromethane, 0 °C, 48 h; (iii) acetonitrile, 80 °C, 2 h; and (iv) ethanol, piperidine, 100 °C, 4 h.

### 3.2 Photophysical Properties and Theoretical Calculations of AIEgens

As illustrated in Figure 3A,B, the AIEgens exhibit absorption in the 400–600 nm region and emission over the 500–850 nm, with the majority of emissions located in the red to near-infrared (NIR) range. SC-3 and SC-4 present comparable absorption maxima at 463 and 466 nm, respectively. Although DFT calculations indicate comparable band gaps for SC-3 and SC-4 in aqueous solution, the hydroxyl group in SC-4 enhances its solubility in water, preventing the formation of large aggregates observed for SC-3 through dynamic light scattering measurement (Figure 4 and Figure S8). Consequently, SC-4 exhibits a slightly red-shifted absorption peak about 3 nm. Additionally, although SC-4 exhibits a similar degree of molecular twisting comparable to that of SC-3, its shorter molecular length of 1.8326 nm enhances its solubility. Furthermore, compared to SC-1 and SC-2, SC-3 possesses the most twisted molecular conformation and the longest molecular length, which facilitates the formation of larger aggregates in aqueous solution and effectively suppresses π–π stacking. This structure more easily enhances both radiative and nonradiative transition rates (Figure 4). Additionally, as shown in Figure 3C and Figure S9, SC-3 exhibits the highest ROS generation capacity, far exceeding that of the other three compounds. Specifically, after continuous white light irradiation for 90 s, the ROS yield of SC-3 is 5.15-fold higher than that of SC-1. DFT calculations further reveal that SC-3 has the smallest singlet–triplet energy gap (Δ*E*_S1T1_) among these AIEgens, calculating only 0.7603 eV, which corroborates the experimental observations, confirming the superior ROS generation capability of SC-3 (Figure S10 and Table S1). Furthermore, according to Kasha’s rule, the Δ*E*_S1T1_ value of SC-3 is below 0.98 eV, making it prone to generating Type-I ROS, with O_2_^–•^ levels exceeding those of OH**·**, and SC-3 also produces ^1^O_2_ under white light irradiation (Figure S11).^43–45^ As illustrated in Figure 3D, SC-3 preferentially generates O_2_^–•^ and OH**·** within MDA-MB-231 cells upon light exposure, whereas ^1^O_2_ generation remains negligible. We further observed that SC-3 localizes predominantly to mitochondria in MDA-MB-231 cells, with a colocalization coefficient of 0.91 (Figure S12). Upon irradiation, the generated ROS primarily disrupt the mitochondrial membrane, thereby triggering activation of the caspase cascade, which in turn markedly upregulates caspase3 expression and initiates the apoptotic pathway in tumor cells (Figure S13).^46–48^

**Figure 3:**
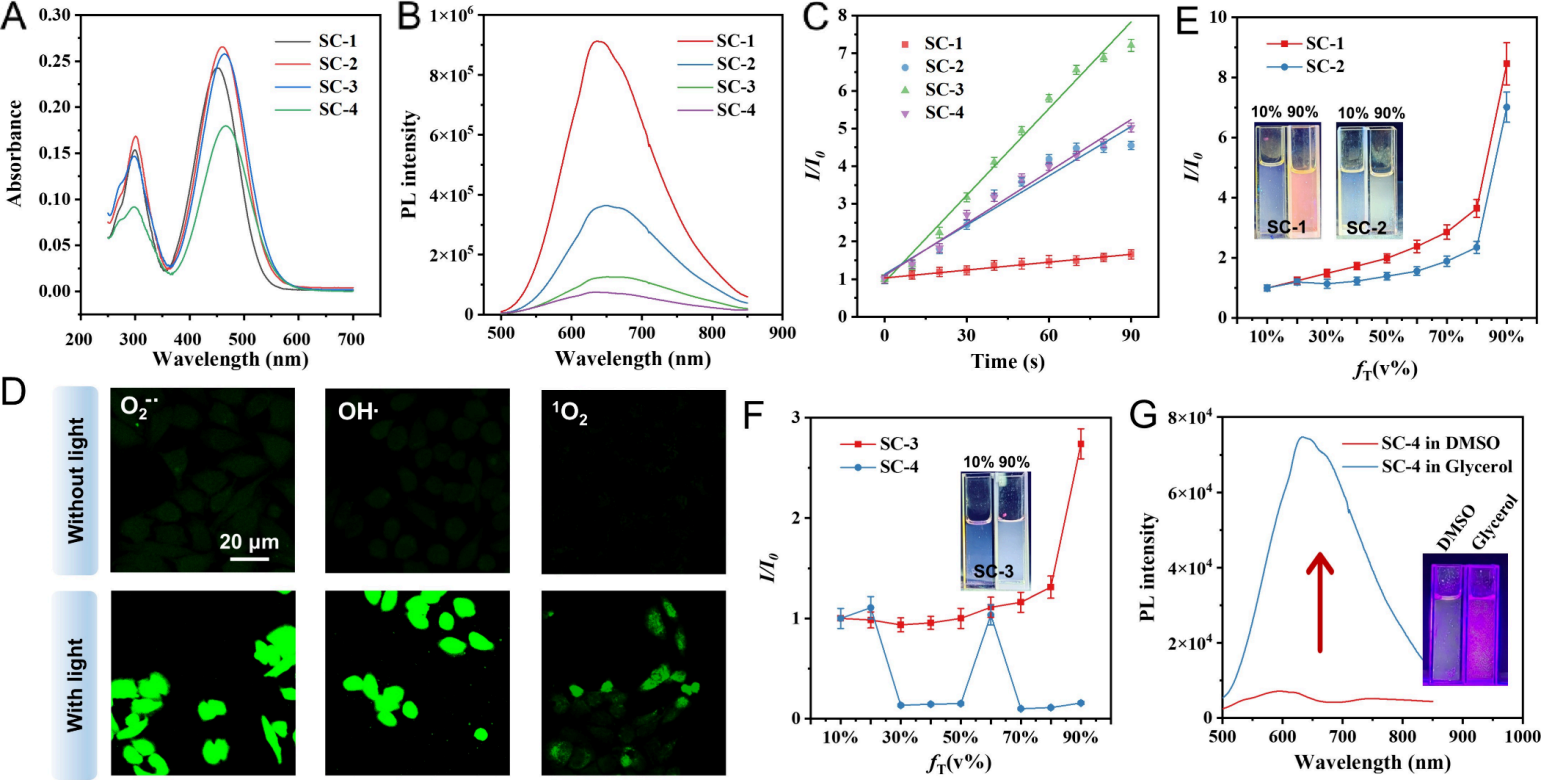
Photophysical properties of the SC-*n* AIEgens. (A, B) Absorption and fluorescence emissions of the SC-*n* compounds in water at a concentration of 10 μM. (C) Quantitative analysis of ROS generation by the SC-*n* compounds under white light irradiation (40 mW/cm^2^). (D) After MDA-MB-231 cells were incubated with SC-3 for 4 h, various ROS probes were added to the culture medium, followed by white light irradiation and 488 nm excitation for confocal laser scanning microscopy imaging. (E) AIE curves of SC-1 and SC-2 in Tol/DMSO solvent mixtures. Inset: fluorescence photographs under 365 nm UV light in mixed solvents containing 10 and 90% Tol. (F) AIE curves of SC-3 and SC-4. Inset: fluorescence photographs of SC-3 in 10 and 90% Tol under 365 nm excitation. (G) Fluorescence emission spectra of SC-4 in DMSO and pure glycerol. Inset: fluorescence images under 365 nm UV light.

**Figure 4:**
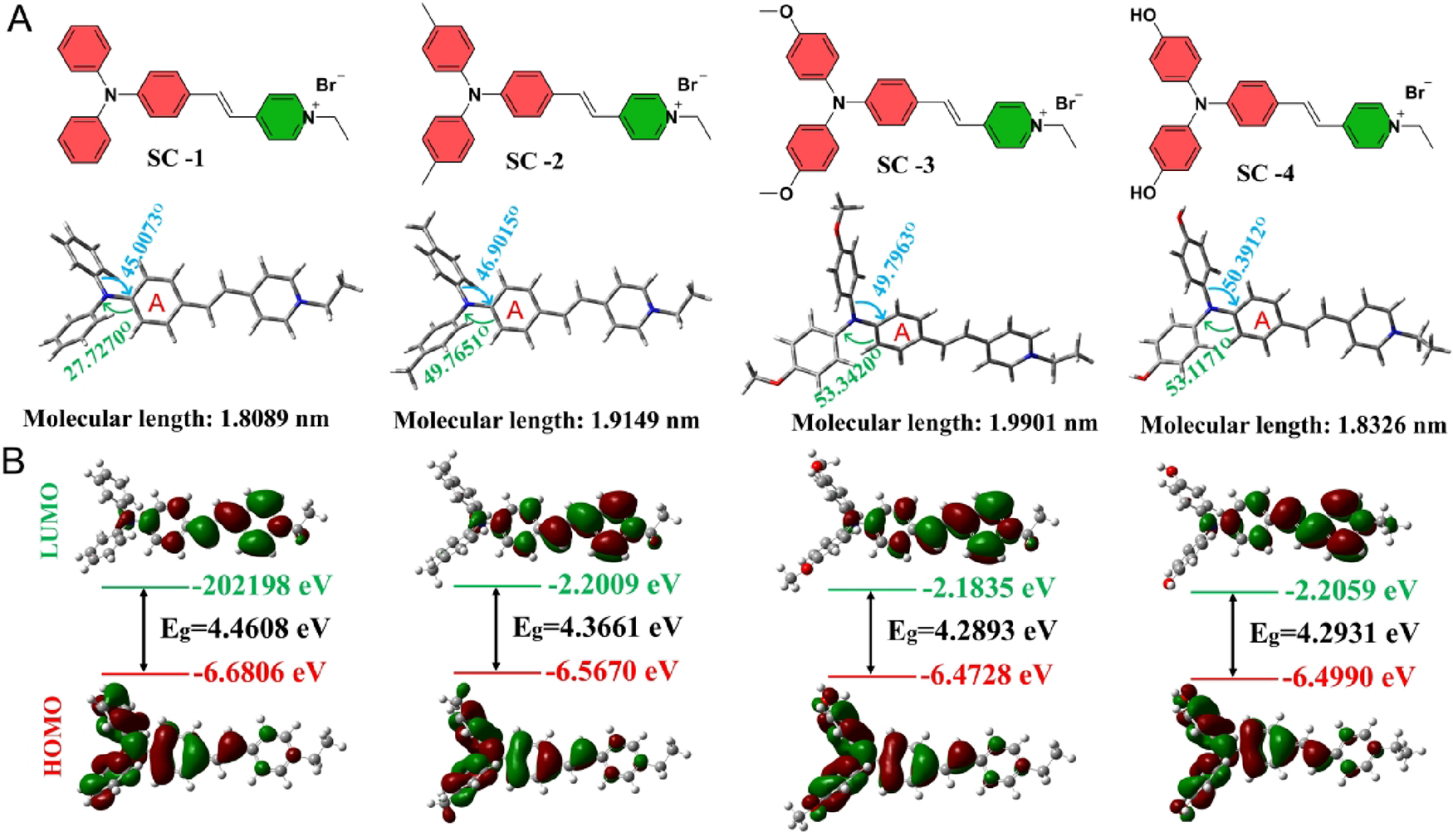
DFT calculations of four SC-*n* AIEgens. (A) Molecular structures of the AIEgens, along with their optimized structures, molecular lengths, and dihedral angles in water. (B) HOMO/LUMO electron cloud distributions and band gaps of the AIEgens.

### 3.3 Luminescence Mechanism of AIEgens

In this study, the Tol/DMSO mixture was selected to evaluate the AIE properties, as the pronounced polarity contrast between the two solvents promotes stable and reproducible aggregation of AIEgens. SC-1, SC-2, and SC-3 exhibit pronounced AIE characteristics in Tol/DMSO mixed solvents as the proportion of the poor solvent Tol gradually increases. In contrast, SC-4, which contains a hydroxyl group introduced at the terminus of the triphenylamine moiety, has no AIE effect (Figure 3E,F and Figure S14). SC-*n* AIEgens exhibit notable solvatochromic behavior in various solvents, indicating a prominent intramolecular charge transfer (ICT) character in their excited states (Figure S15). Numerous studies have shown that ICT process serves as a key mechanism underpinning their transitions. The observed variations in fluorescence intensity of SC-4 in the mixed Tol/DMSO solvent likely arise from several factors; the hydroxyl group increases the polarity of SC-4 and enhances D–A charge transfer (CT). In the presence of DMSO, the excited states of the molecules tend to form twisted intramolecular charge transfer (TICT) states or strongly stabilized CT states. These intermediate states are stabilized in polar solvents, which enhances nonradiative decay pathways and quenches fluorescence, thereby masking the AIE effect. Additionally, the hydroxyl groups can form hydrogen bonds with DMSO, acting as an effective hydrogen bond acceptor, which increases the solubility of the molecules in the mixed solvent and inhibits the formation of hydrophobic, tightly packed aggregates. In other words, favorable solvent-molecule interactions prevent the formation of compact aggregates. Restriction of intramolecular motion (RIM) is a key factor driving the AIE phenomenon, as its suppression enhances fluorescence intensity and AIE characteristics. Specifically, as shown in Figure 3G, the fluorescence intensity of SC-4 dispersed in glycerol is approximately 10.36 times higher than that in DMSO. Therefore, it is also an excellent AIE material. Based on the above considerations, introducing hydrophobic methoxy groups at the terminus of the triphenylamine moiety enables more direct regulation of molecular hydrophilicity, D-π-A strength, and molecular twisting. The resulting SC-3 exhibits stronger AIE behavior in aqueous environments, making it more suitable for cellular imaging and tumor therapy.

### 3.4 Synthesis of Supramolecular 2SC-3/CB[8] Photosensitizers

CB­[8] has a polycarbonyl structure, wherein the carbon atoms of the carbonyl groups bear partial positive charges while the oxygen atoms carry partial negative charges (Figure S16A). The electron cloud around these groups is relatively stable, making CB[8] unlikely to readily lose or accept electrons to form long-lived radicals. Thus, it is not a typical photosensitizing electron donor or acceptor.^49,50^ However, the carbonyl oxygen atoms can act as weak Lewis bases and engage in electrostatic or dipole interactions with positively charged or electron-deficient sites. Therefore, when quaternary ammonium-type AIE molecules form the supramolecular photosensitizer 2SC-3/CB[8], CB[8] acts as a neutral, cage-like host that provides a hydrophobic cavity, electrostatic shielding, and conformational restriction (Figure 5A). These effects collectively restrict intramolecular motions, induce intermolecular π–π and CT interactions, and suppress disordered aggregation, thereby significantly enhancing ISC efficiency and triplet-state yield, which in turn improves the generation efficiency of ^1^O_2_.^41,51,52^ As shown in Figure 5B and Figure S16B, the Job’s plot indicates that SC-3 and CB[8] assemble in the 2:1 stoichiometric ratio. Moreover, due to the shielding effect of CB[8], the proton signals of the ethyl groups in SC-3 exhibit an upfield shift about 0.223 ppm (Figure S17). The binding constant between SC-3 and CB[8] was determined to be 1.39 × 10^5^ M^–1^, further confirming the formation of the supramolecular photosensitizer (Figure 5C). The tightly packed π–π stacking and ICT interactions within the CB[8] cavity in 2SC-3/CB[8] significantly reduce the highest occupied molecular orbital/lowest unoccupied molecular orbital (HOMO/LUMO) bond gap. Additionally, the CB[8] microenvironment stabilizes the excited states in polar solvent, further lowering the excited-state energy. Consequently, the absorption and emission spectra of 2SC-3/CB[8] exhibit pronounced red shifts (Figure 5D,E). Furthermore, while SC-3 forms nanoparticles with an average diameter of 76.62 ± 8.61 nm, the presence of CB[8] suppresses disordered aggregation, resulting in smaller nanoparticles with an average diameter of 53.24 ± 6.21 nm. The polydispersity index (PDI) of 0.23 further indicates a relatively uniform distribution of the nanosupramolecular photosensitizer 2SC-3/CB[8] (Figure 5F and Figure S8). The Zeta potential decreases to −12.36 ± 2.40 mV for 2SC-3/CB[8], suggesting relatively high biocompatibility during blood circulation (Figure 5G). Finally, consistent with our hypotheses, the construction of the supramolecular photosensitizer 2SC-3/CB[8] enhances ^1^O_2_ generation, as validated at both chemical and cellular levels (Figure 5H and Figures S18 and S19). Numerous studies have shown that when one CB[8] encapsulates two photosensitizer molecules, the resulting supramolecular complex tends to be more stable and exhibit optimal photosensitizing properties.^41,49,51^ While we systematically investigate the properties of the nanosupramolecular photosensitizer 2SC-3/CB[8] in this study, the performance of supramolecular photosensitizers formed at other assembly ratios has not yet been explored, providing a valuable direction for further optimization and extension in future research.

**Figure 5:**
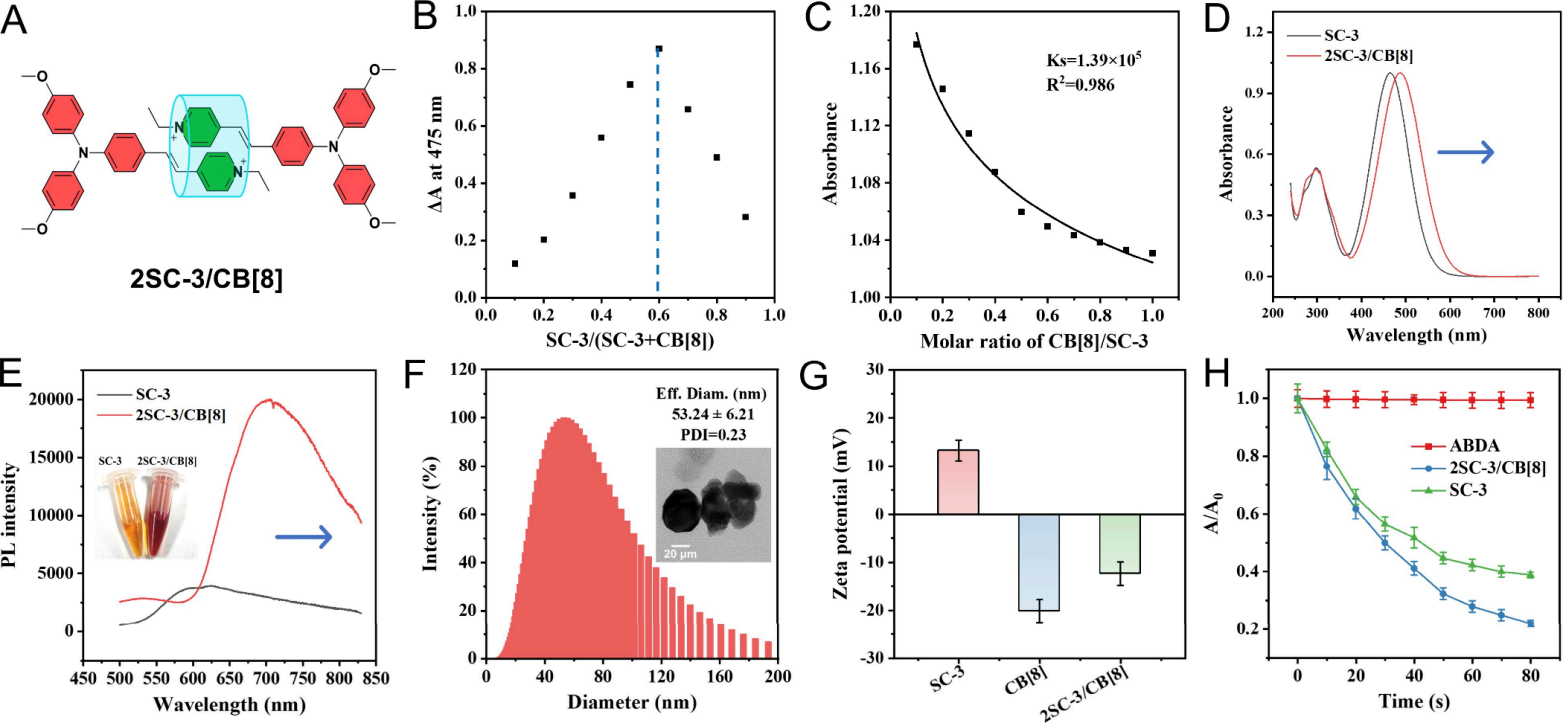
Synthesis and characterization of nanosupramolecular photosensitizer 2SC-3/CB[8]. (A) Schematic chemical structure of the assembled 2SC-3/CB[8]. (B) Job’s plot of SC-3 and CB[8] indicates a 2:1 stoichiometric ratio. (C) Binding constant between SC-3 and CB[8] in 2SC-3/CB[8]. (D) Absorption spectra of SC-3 and 2SC-3/CB[8] in water at a concentration of 10 μM. (E) Emission spectra and naked eye images of SC-3 and 2SC-3/CB[8] in water at a concentration of 10 μM. (F) Particle size distribution and transmission electron microscopy (TEM) imaging picture of 2SC-3/CB[8] at a concentration of 10 μM. The average diameter is 53.24 ± 6.21 nm, PDI = 0.23. (G) Zeta potentials of SC-3, CB[8], and 2SC-3/CB[8] samples at a concentration of 10 μM. (H) Level of ^1^O_2_ generation by SC-3 and 2SC-3/CB[8] at same SC-3 concentration, detected using an ABDA (10 μM) indicator.

### 3.5 Cytotoxicity Test

Previous studies have shown that ROS-mediated tumor therapies are highly diverse. In addition to drugs or photosensitizers that induce tumor cell death through direct ROS accumulation, stimulus-responsive photosensitizers designed according to tumor microenvironment characteristics have achieved remarkable progress in precise tumor-targeted treatment.^53–56^ Moreover, supramolecular regulation strategies based on host–guest interactions have demonstrated broad potential for controlling ROS species, offering new ideas and strategies for the development of next-generation precision theranostics for tumor.^57–62^ Therefore, considering the hypoxic tumor microenvironment and the oxygen consumption required for ^1^O_2_ generation, we adopted a combined therapeutic strategy integrating PDT using 2SC-3/CB[8] with the hypoxia-activated prodrug TPZ to achieve efficient utilization of ROS (Figure S20). The key aspect of this approach lies in leveraging the hypoxia induced by oxygen depletion during ^1^O_2_ generation to enhance TPZ activity. As demonstrated in Tables S2–S4 and Figure 6A, the cytotoxicity of TPZ increases with the degree of hypoxia in the culture environment. In contrast, the phototoxic effects of SC-3 and 2SC-3/CB[8] on MDA-MB-231 cells gradually diminish under more severe hypoxic conditions, attributable to reduced oxygen availability impairing ^1^O_2_ production and thereby decreasing compound-induced cytotoxicity. When TPZ is combined with PDT, the antitumor efficacy markedly surpasses that of either TPZ alone or PDT alone. Notably, under 2% oxygen conditions, the combined treatment exhibits a significantly reduced IC_50_ of 0.42 ± 0.064 μM, which decreased by nearly 98.13% compared with TPZ alone (Table S4 and Figure 6B). Even without phototherapy, under 2% O_2_ conditions, the IC_50_ value of 2SC-3/CB[8] + TPZ decreases by 17.25% compared with TPZ alone, indicating that CB[8] also exerts a synergistic effect in promoting tumor cell death (Table S4). Furthermore, the reagents employed in this study, such as TPZ, CB[8], SC-3, and 2SC-3/CB[8], demonstrate high safety profiles, causing negligible cytotoxicity to HUVECs (Table S5).

**Figure 6:**
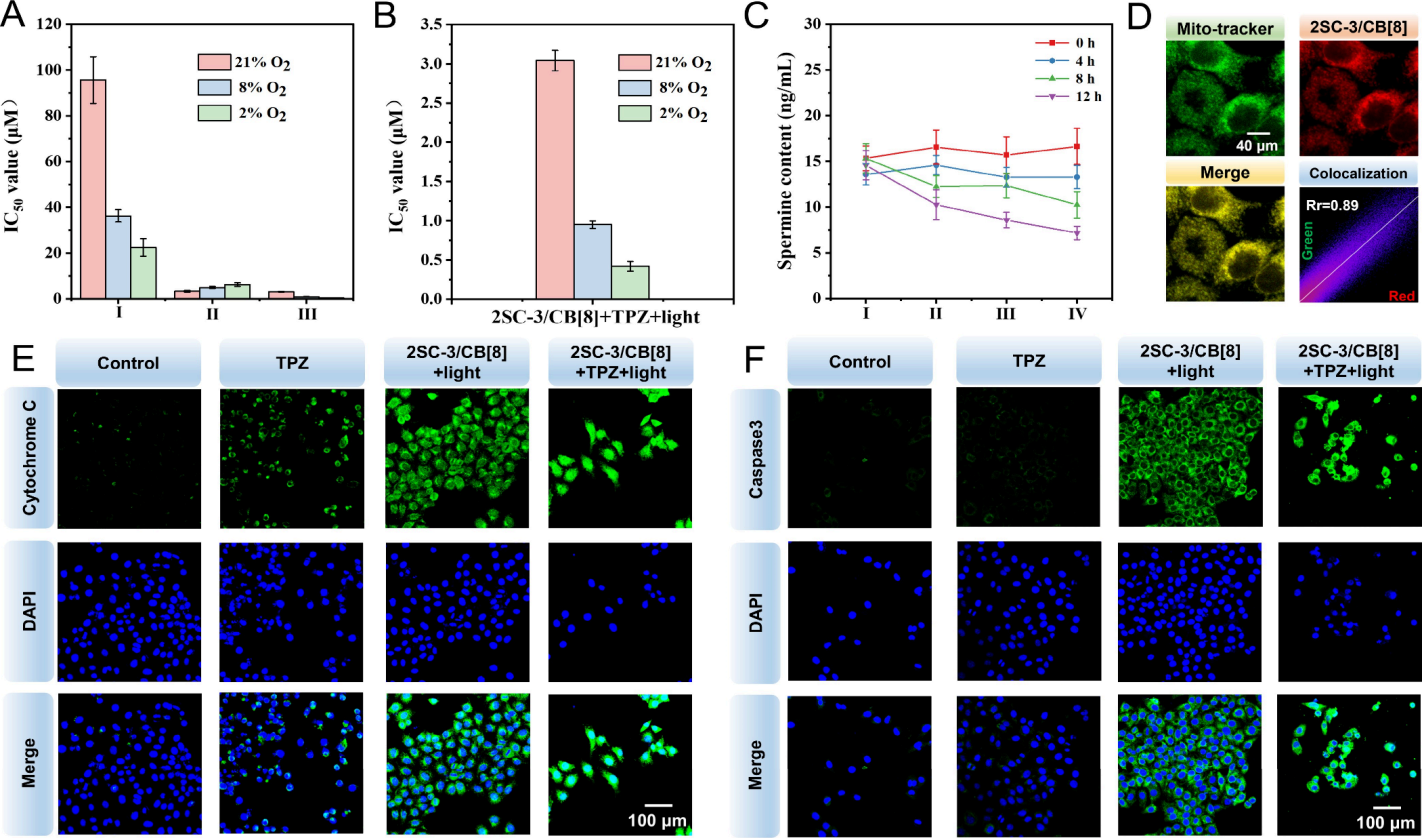
Combined tumor therapy using 2SC-3/CB[8] and TPZ. (A) IC_50_ values of different treatment groups under varying O_2_ content (I: TPZ; II: 2SC-3/CB[8] + light; III: 2SC-3/CB[8] + TPZ + light). (B) Specific IC_50_ values of 2SC-3/CB[8] + TPZ + light group under 2, 8, and 21% O_2_ concentrations. (C) Quantification of spermine levels in MDA-MB-231 cells after treatment with different substances over time (I: Control; II: CB[8]; III: 2SC-3/CB[8]; IV: 2SC-3/CB[8] + TPZ). (D) Colocalization analysis of 2SC-3/CB[8] at a concentration of 10 μM with mitochondria in MDA-MB-231 cells. (E, F) Immunofluorescence images of cytochrome c and caspase3 in MDA-MB-231 cells after different treatments. The concentration of the compound is 10 μM, white light power is 40 mW/cm^2^, and the illumination time is 20 min.

### 3.6 Antitumor Mechanism of 2SC-3/CB[8] + TPZ + Light

Spermine generally exhibits pro-tumorigenic effects in cancers, and its levels are significantly elevated in various tumors, where it stabilizes DNA and promotes transcription and translation, thereby accelerating cell proliferation.^63,64^ Consequently, spermine serves both as a potential tumor biomarker and as a therapeutic target in polyamine metabolism-based treatments. Moreover, spermine carries multiple positive charges in aqueous solution, enabling strong electrostatic attraction with the carbonyl oxygen atoms at the portal of CB[8]. Its hydrophobic carbon chain can insert into the hydrophobic cavity of CB[8], reducing water contact, while the amino groups form hydrogen bonds with the carbonyls, further stabilizing the complex.^65,66^ As demonstrated in Figure 6C and consistent with previous studies, during tumor treatment combining PDT of 2SC-3/CB[8] with TPZ, the concentration of free spermine significantly decreases over time, thereby inhibiting tumor growth.^63,65–68^ The nanosupramolecular photosensitizer 2SC-3/CB[8], formed by encapsulating quaternary ammonium salts within CB[8], possesses size and morphology suitable for penetrating cellular and mitochondrial membranes. The rigid, cage-like structure of CB[8] constrains the conformation of the quaternary ammonium salts, concentrating positive charges on the surface and strengthening electrostatic interactions with the negatively charged mitochondrial membrane. Consequently, 2SC-3/CB[8] exhibits a high index of mitochondrial colocalization with the colocalization coefficient of 0.89. Compared with the higher mitochondrial targeting coefficient of SC-3 alone, the assembly with CB[8] may slightly modulate the interaction of SC-3 with the mitochondrial membrane, but this does not compromise its targeting ability. On one hand, ROS generated during PDT markedly deplete intracellular glutathione (GSH), concomitant with significant upregulation of glutathione disulfide (GSSG). The malondialdehyde (MDA) level in cells reaches as high as 4.07 ± 1.07 μmol/L, indicating pronounced oxidative stress under this therapy modality (Figure S21). ROS release also damages the mitochondrial membrane, subsequently activating the cytochrome c/caspase9/caspase3 apoptotic pathway and thereby initiating tumor cell apoptosis (Figure 6E,F and Figure S22). Additionally, TPZ, as a chemotherapeutic agent, inflicts damage on the cell nucleus, which is essential for the full activation of apoptotic pathway. Accordingly, in cell migration assays, the combined treatment of 2SC-3/CB[8] + TPZ + light significantly suppresses tumor cell migration, further evidencing its potent therapeutic efficacy (Figure S23).

## 4 Conclusions

In summary, we synthesized four SC-*n* AIEgens and identified SC-3 as the most potent photosensitizer through DFT calculations and experimental validation, elucidating its mitochondrial-mediated apoptotic mechanism. Compared with SC-1, the ROS generation capability of SC-3 increased by 5.15 times. To further enhance efficacy, we developed the nanosupramolecular photosensitizer 2SC-3/CB[8], which significantly increases ^1^O_2_ generation by 35% compared with SC-3 alone, and exacerbates tumor hypoxia. Under these conditions, combined treatment with 2SC-3/CB[8] and the hypoxia-activated chemotherapeutic TPZ markedly promoted MDA-MB-231 cell death and enhanced TPZ’s antitumor efficacy. Additionally, CB[8]-mediated spermine depletion contributed to tumor cell killing. Overall, the rational design of 2SC-3/CB[8] and its synergistic application with TPZ represents a promising strategy for solid tumor therapy.

## Supplementary Material



## Notes

Detailed information on the specific synthesis route and characterization data, spectral data, and theoretical calculation data of the target compounds; images of target protein immunofluorescence and tumor cell migration; and data on the toxicity of different compounds (PDF)
