# Supplementary material for: Supramolecular Aggregation-Induced Emission Photosensitizer Encapsulated by Cucurbit[8]uril Nanocavity Boosts Hypoxia-Activated Tumor Therapy
Source: ACS Appl Nano Mater. 2025 Oct 18;8(43):21009–18. doi: 10.1021/acsanm.5c03879 (PMC12584102; doi:10.1021/acsanm.5c03879)
Supplement: Supplementary file 1 [file an5c03879_si_001.pdf]

Supporting Information

Supramolecular Aggregation-Induced Emission Photosensitizer Encapsulated by Cucurbit[8]uril Nanocavity Boosts Hypoxia-Activated Tumor Therapy

Wen Liu,[a,†] Zeyu Zhang,[a,†] Qing Huang,[a] Rishi Pai,[a] Lucas Liu,[a] Zufeng Ding,[b] Xing Wang,[a,] Zhicheng Jin[a,*]*

[a] Department of Chemistry, Georgia State University, Atlanta, Georgia 30303, United States

[b] Department of Biology, Georgia State University, Atlanta, Georgia 30303, United States

† Authors contributed to the work equally.

* Corresponding author's email: zjin4@gsu.edu (Z.J.), xwang82@gsu.edu (X.W.)

1. Experimental methods

Synthesis and characterization of target compounds

Synthesis and characterization a

Compound 4-methoxy-*N*-(4-methoxyphenyl)-*N*-phenylaniline (5 g) was dissolved in N,N-dimethylformamide (DMF) solution. After the mixture was stirred evenly, $POCl_3$ (10 mL) was added to it, and the reaction was carried out at 80°C for 24 h. Then the reaction solution was cooled to room temperature, followed by the addition of methanol (MeOH) to quench the $POCl_3$. Finally, all solvents were removed, and the crude product was washed with petroleum ether (PE) as the eluent to obtain a pale-yellow product a with a yield of 55%. ^{1}H NMR (600 MHz, DMSO-d_6) δ 9.70 (s, 1H), 7.65 (d, *J* = 8.7 Hz, 2H), 7.21 (d, *J* = 8.8 Hz, 4H), 7.00 (d, *J* = 8.8 Hz, 4H), 6.68 (d, *J* = 8.7 Hz, 2H), 3.77 (s, 6H).

Synthesis and characterization of b

Compound a (1 g) was dissolved in dichloromethane (DCM), and an appropriate amount of BBr_3 (5 mL) was added to it, and the reaction was continued at 0 °C for 48 h. Upon completion, the solvent was removed to give a black oily crude product. The crude product was further purified by elution with a petroleum ether/ethyl acetate (PE/EA) = 10/1 mixture to afford a pale-yellow solid b in 48% yield. ^{1}H NMR (600 MHz, DMSO-d_6) δ 9.70 (s, 1H), 7.65 (d, *J* = 8.6 Hz, 1H), 7.21 (d, *J* = 8.7 Hz, 2H), 7.14 (d, *J* = 8.3 Hz, 4H), 7.00 (d, *J* = 8.7 Hz, 2H), 6.78 (d, *J* = 8.3 Hz, 3H), 6.69 (d, *J* = 8.6 Hz, 1H).

Synthesis and characterization of c

4-Methylpyridine (5 g) and 2-bromoethane (3 g) were dissolved in acetonitrile (ACN) solution and reacted at 80 °C for 4 h. Upon completion, the reaction solvent was removed to obtain a pink solid. The resulting crude product was then washed with diethyl ether to afford pink-white solid c in 75% yield. ^{1}H NMR (600 MHz, DMSO-d_6) δ 8.94 (d, *J* = 6.0 Hz, 2H), 7.95 (d, *J* = 6.1 Hz, 2H), 4.58 (q, *J* = 7.3 Hz, 2H), 3.73 (s, 3H), 1.50 (t, *J* = 7.3 Hz, 3H).

Synthesis and characterization of SC-n

As an example, for the synthesis of compound SC-1, d-1 (1 mmol) and c (1 mmol) were dissolved in ethanol. After thorough stirring, piperidine (0.1 mmol) was added to the mixture, and the reaction was continued at 100 °C for 4 h. The solvent was then removed from the reaction mixture to obtain a crude product, which was purified by elution with a DCM/MeOH (30/1) mixed solvent to afford the red solid product SC-1 in 52% yield. ^{1}H NMR (600 MHz, DMSO-d_6) δ 8.89 (d, *J* = 6.7 Hz, 2H), 8.17 (d, *J* = 6.7 Hz, 2H), 7.97 (d, *J* = 16.2 Hz, 1H), 7.63 (d, *J* = 8.6 Hz, 2H), 7.39 (t, *J* = 7.8 Hz, 4H), 7.32 (d, *J* = 16.2 Hz, 1H), 7.17 (t, *J* = 7.4 Hz, 2H), 7.12 (d, *J* = 7.9 Hz, 4H), 4.50 (q, *J* = 7.2 Hz, 2H), 1.52 (t, *J* = 7.3 Hz, 3H). ^{13}C NMR (151 MHz, DMSO-d_6) δ 153.62, 149.95, 146.67, 144.20, 141.12, 130.33, 130.16, 128.46, 125.87, 124.99, 123.72, 121.18, 120.94, 55.48, 16.64. HR-MS (m/z) (ESI): calcd for $C_{27}H_{25}BrN_2^+$ [M - Br]$^+$: 377.2012; found : 377.2022. Following the above procedure, SC-2 was obtained as a brownish-red solid in 53% yield. ^{1}H NMR (600 MHz, DMSO-d_6) δ 8.89 (d, *J* = 6.9 Hz, 2H), 8.16 (d, *J* = 6.9 Hz, 2H), 7.96 (d, *J* = 16.2 Hz, 1H), 7.59 (d, *J* = 8.8 Hz, 2H), 7.28 (d, *J* = 16.2 Hz, 1H), 7.19 (d, *J* = 8.2 Hz, 4H), 7.02 (d, *J* = 8.3 Hz, 4H), 6.87 (d, *J* = 8.8 Hz, 2H), 4.50 (q, *J* = 7.3 Hz, 2H), 2.30 (s, 6H), 1.51 (t, *J* = 7.3 Hz, 3H). ^{13}C NMR (151 MHz, DMSO-d_6) δ 153.72, 150.35, 144.14, 144.08, 141.33, 134.40, 130.82, 130.15, 127.54, 126.13, 123.57, 120.32, 119.86, 55.39, 22.63, 20.96, 16.65. HR-MS (m/z) (ESI): calcd for $C_{29}H_{29}BrN_2^+$ [M - Br]$^+$: 405.2325; found : 405.2330. SC-3 as a dark-red solid in 56% yield. ^{1}H NMR (600 MHz, DMSO-d_6) δ 8.88 (d, *J* = 6.7 Hz, 2H), 8.14 (d, *J* = 6.7 Hz, 2H), 7.95 (d, *J* = 16.1 Hz, 1H), 7.56 (d, *J* = 8.7 Hz, 2H), 7.24 (d, *J* = 16.1 Hz, 1H), 7.13 (d, *J* = 8.8 Hz, 4H), 6.98 (d, *J* = 8.8 Hz, 4H), 6.74 (d, *J* = 8.7 Hz, 2H), 4.49 (q, *J* = 7.2 Hz, 2H), 3.77 (s, 6H), 1.51 (t, *J* = 7.3 Hz, 3H). ^{13}C NMR (151 MHz, DMSO-d_6) δ 153.45, 148.73, 148.26, 144.54, 144.48, 138.86, 134.91, 134.52, 133.74, 130.72, 130.57, 127.19, 125.58, 124.91, 124.36, 123.71, 121.17, 56.96, 22.63, 20.92. HR-MS (m/z) (ESI): calcd for $C_{29}H_{29}BrN_2O_2^+$ [M - Br]$^+$: 437.2224; found : 437.2224. SC-4 as a black solid in 46% yield. ^{1}H NMR (600 MHz, DMSO-d_6) δ 9.51 (s, 2H), 8.84 (d, *J* = 6.1 Hz, 2H), 8.11 (d, *J* = 6.1 Hz, 1H), 7.91 (d, *J* = 16.0 Hz, 1H), 7.52 (d, *J* = 8.4 Hz, 2H), 7.19 (d, *J* = 16.2 Hz, 1H), 7.03 (d, *J* = 8.2 Hz, 4H), 6.80 (d, *J* = 8.3 Hz, 4H), 6.66

(d, J = 8.3 Hz, 2H), 5.76 (s, 1H), 4.47 (dd, J = 13.4, 6.4 Hz, 2H), 1.50 (t, J = 6.8 Hz, 3H). ^{13}C NMR (151 MHz, DMSO-d_6) δ 155.61, 153.90, 151.66, 143.95, 141.80, 137.64, 130.24, 128.82, 128.61, 125.50, 123.26, 118.99, 116.91, 116.70, 55.40, 16.64. HR-MS (m/z) (ESI): calcd for $C_{27}H_{25}BrN_2O_2^+$ [M - Br]$^+$: 409.1911; found : 409.1919.

Cell culture

In this study, the human triple-negative breast cancer cell line MDA-MB-231 was cultured in a 5% CO_2 atmosphere at 37 °C. The culture medium used was Dulbecco's Modified Eagle Medium (DMEM) complete medium, consisting of 5% fetal bovine serum, 93% DMEM incomplete medium, and 2% penicillin–streptomycin solution. Tumor cell thawing, passaging, and cryopreservation were performed according to conventional methods. The CO_2 concentration in the incubator could be adjusted as needed according to experimental requirements. Human umbilical vein endothelial cells (HUVECs) were also cultured and subjected to relevant experiments using this method.

Immunofluorescence imaging

MDA-MB-231 cells in the logarithmic growth phase were seeded into confocal culture dishes. After the cells had fully adhered, they were treated under different conditions, and the culture medium was removed subsequently. Then, these cells were fixed with 4% paraformaldehyde, permeabilized with 0.1% Triton-100, and blocked with 5% bovine serum albumin (BSA), followed by multiple washes with phosphate buffer solution (PBS). Finally, the primary antibody and fluorescence-labeled secondary antibody were sequentially incubated with the cells, after which the nuclear dye DAPI was added to it for confocal laser scanning microscopy (CLSM) imaging. The expression of cytochrome c, caspase9, and caspase3 in the cells was evaluated using the same procedure.

Imaging of different types of reactive oxygen species (ROS) within cells

SC-3 at a concentration of 10 μM was added to MDA-MB-231 cells and co-incubated for 4 h, followed by the addition of 10 μM dihydrorhodamine-123 (DHR-123), hydroxyphenyl fluorescein (HPF), and singlet oxygen sensor green (SOSG),

respectively. After further 15 min of co-incubation, the cells were irradiated with white light (40 mW/cm^2, 5 min) and imaged using CLSM to detect different types of ROS in the tumor cells. In parallel, tumor cells without light irradiation were also subjected to CLSM imaging for detection. The SOSG level of 2SC-3/CB[8] under illumination is also detected using this method.

Cell migration experiment

MDA-MB-231 cells were seeded in a six-well plate and cultured in complete medium. When the cells reached approximately 90% confluence, a scratch was made across the bottom of the well. The six-well plate was then washed with PBS to remove detached cells and the remaining cells were cultured in incomplete medium. After the remaining cells were co-incubated with different compounds (10 μM) for 4 h followed by exposure to white light (40 mW/cm^2) of 20 min and continued culturing for 24 h, the bottom of the six-well plate was photographed subsequently. Notably, the group without light only received drug treatment for 24 h, and the different drug concentrations for combined administration are all 10 μM.

Detection of intracellular substances

The contents of glutathione (GSH), oxidized glutathione (GSSG), and malondialdehyde (MDA) were determined using the ultraviolet (UV) spectrophotometric method. In brief, a standard curve was prepared from standard substances, and the absorbance of cell extracts-denatured by adding different reagents-was measured and substituted into the standard curve to further estimate the content or activity of the target substances. The contents of the target substances in different treatment groups were analyzed for significant differences. The spermine in tumor cells was detected following the steps in the enzyme-linked immunosorbent assay kit.

Data analysis

Data are represented as mean ± standard deviation (S.D.), except for special clarification. Statistical comparisons were made by two-tailed unpaired Student's t-test for two groups. P values of < 0.05 and < 0.01 were considered statistical difference and statistically significant difference, respectively, * $P < 0.05$, ** $P < 0.01$.

2. Experimental data

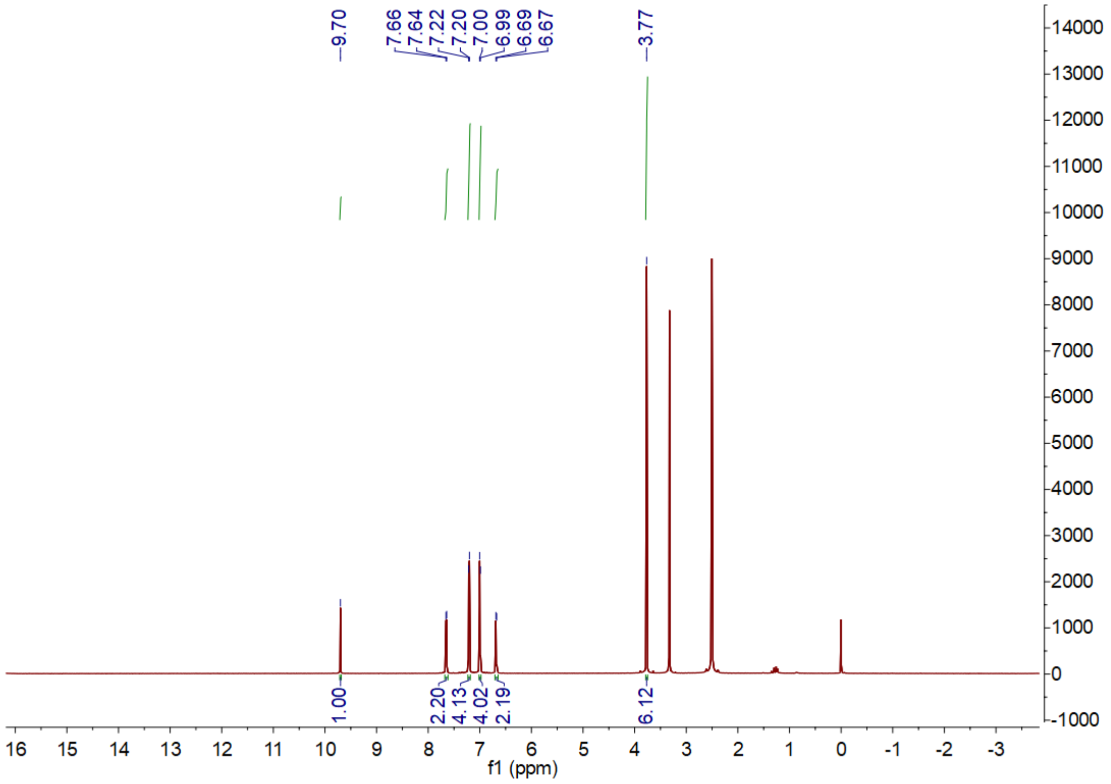

Figure S1. ^{1}H NMR (600 MHz, DMSO-d_6) spectra of a.

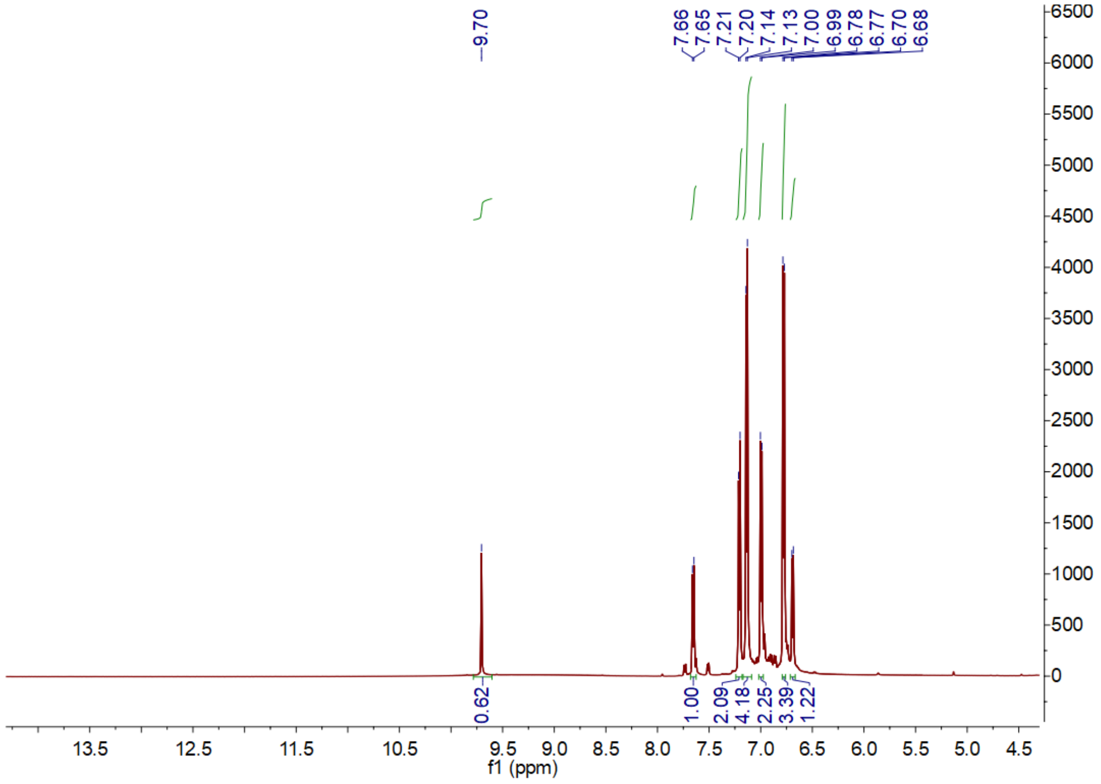

Figure S2. ^{1}H NMR (600 MHz, DMSO-d_6) spectra of b.

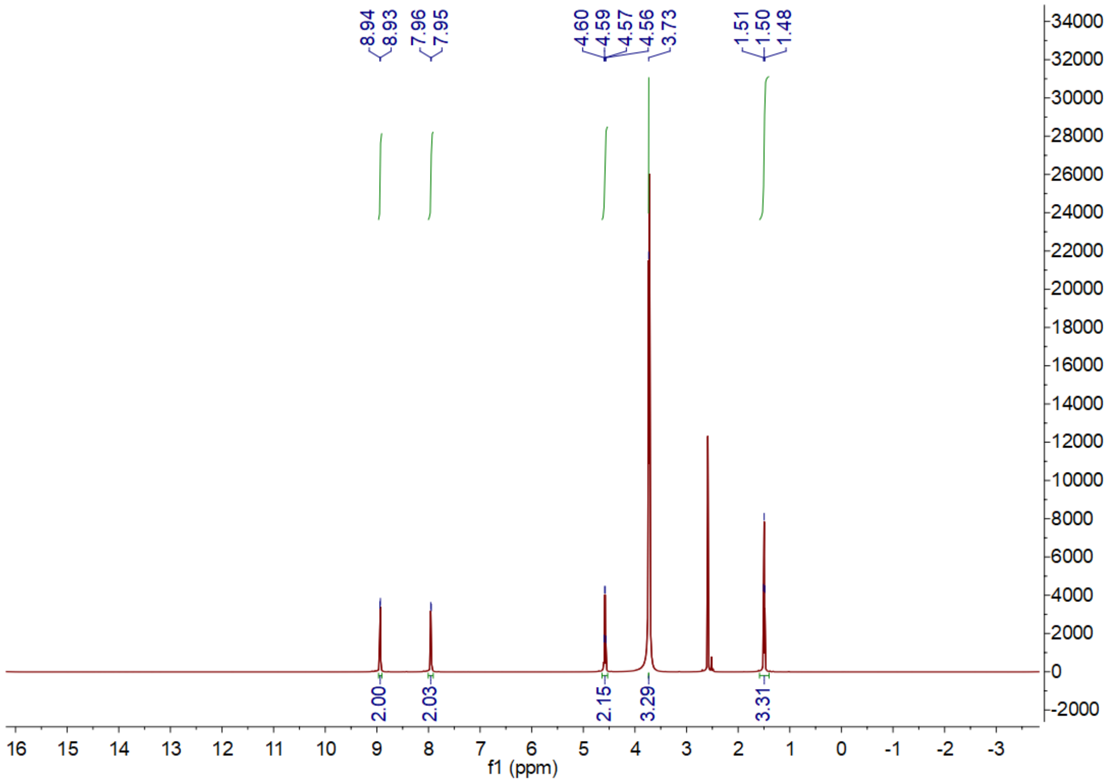

Figure S3. ^{1}H NMR (600 MHz, DMSO-d_6) spectrum of c.

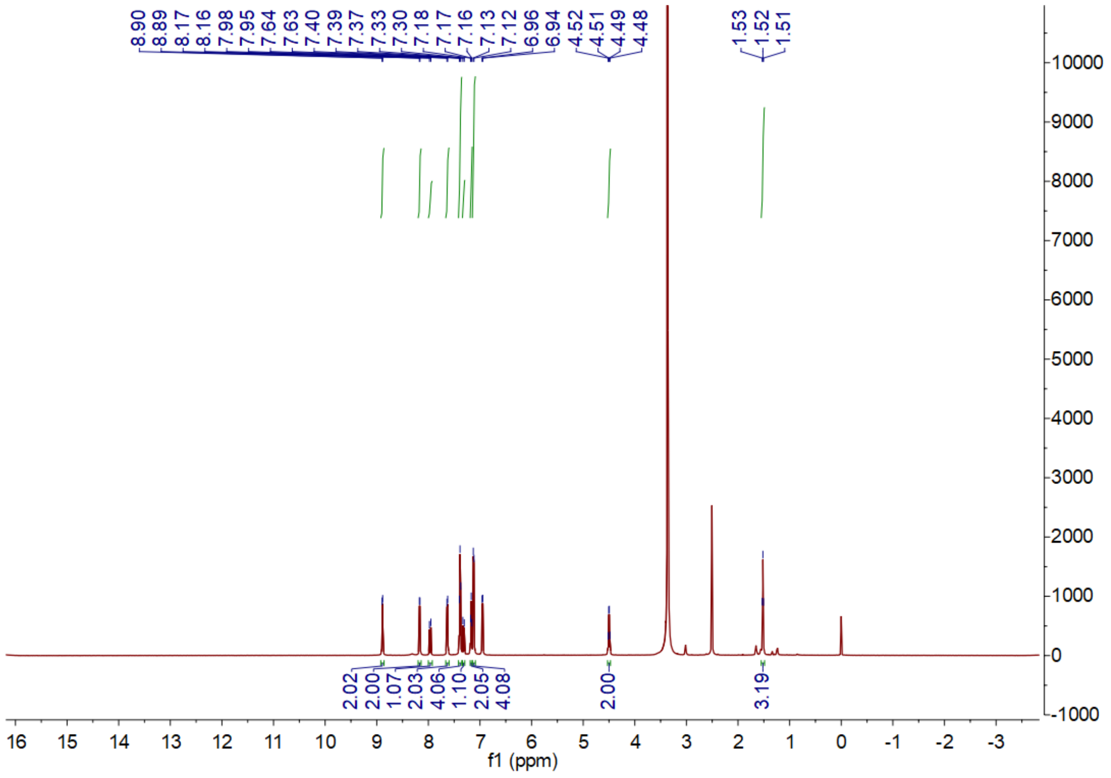

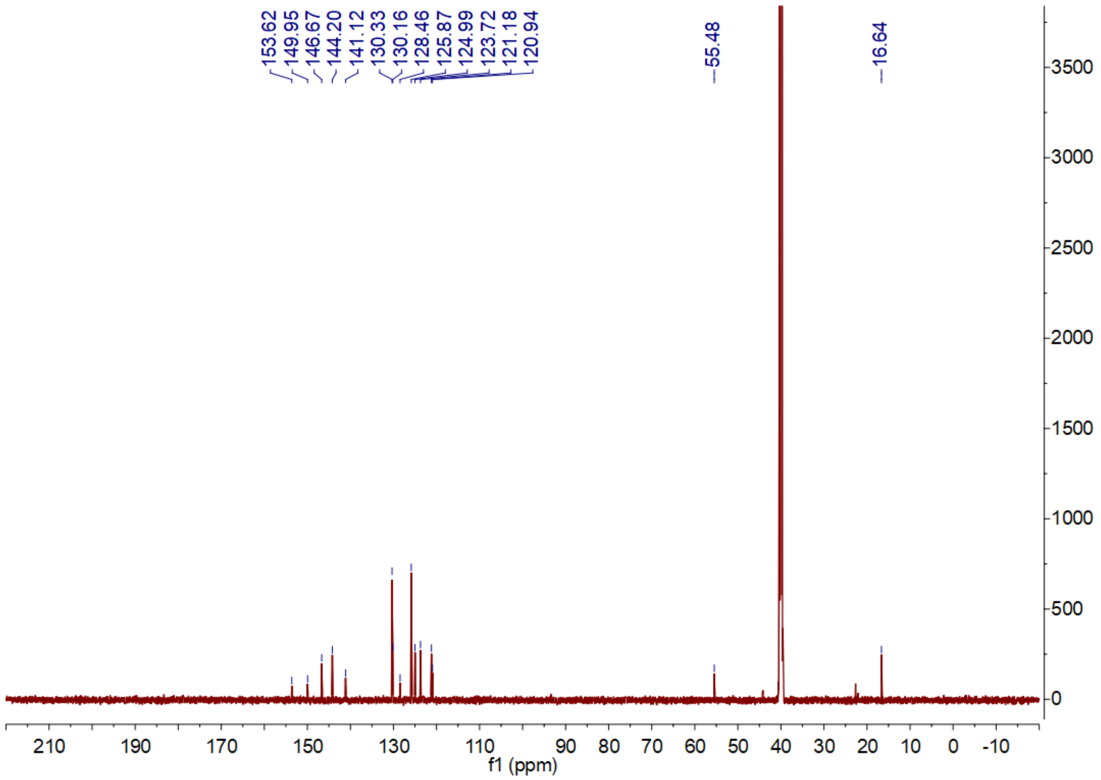

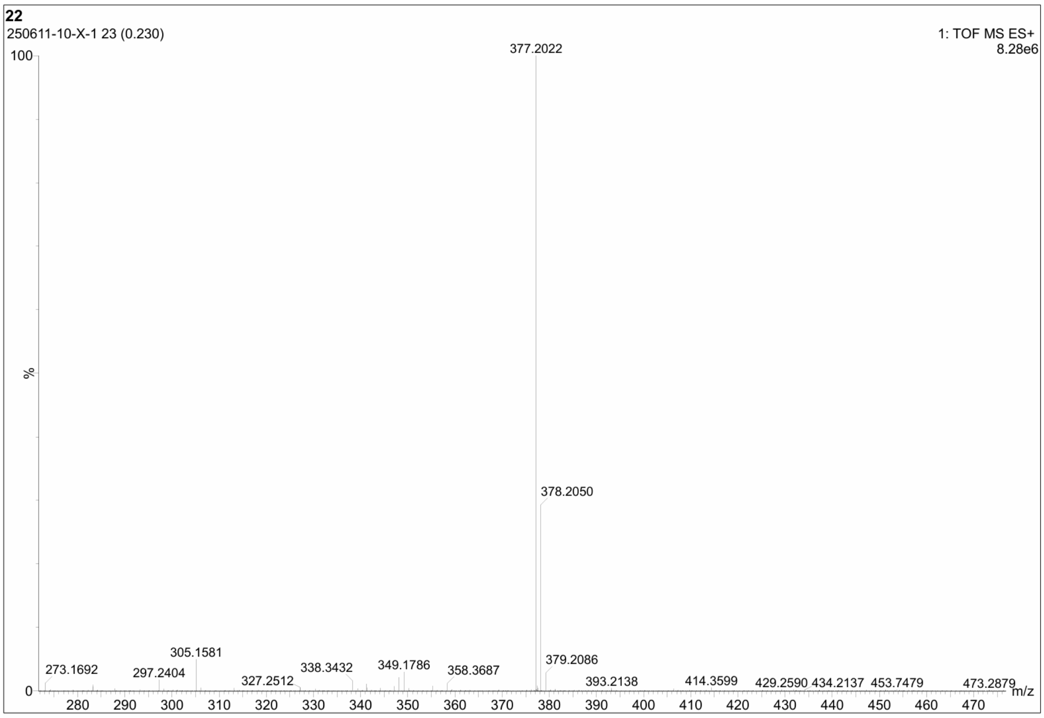

Figure S4. ^{1}H NMR (600 MHz, DMSO-d_6), ^{13}C NMR (151 MHz, DMSO-d_6) and HR-MS (ESI) (positive mode) spectra of SC-1.

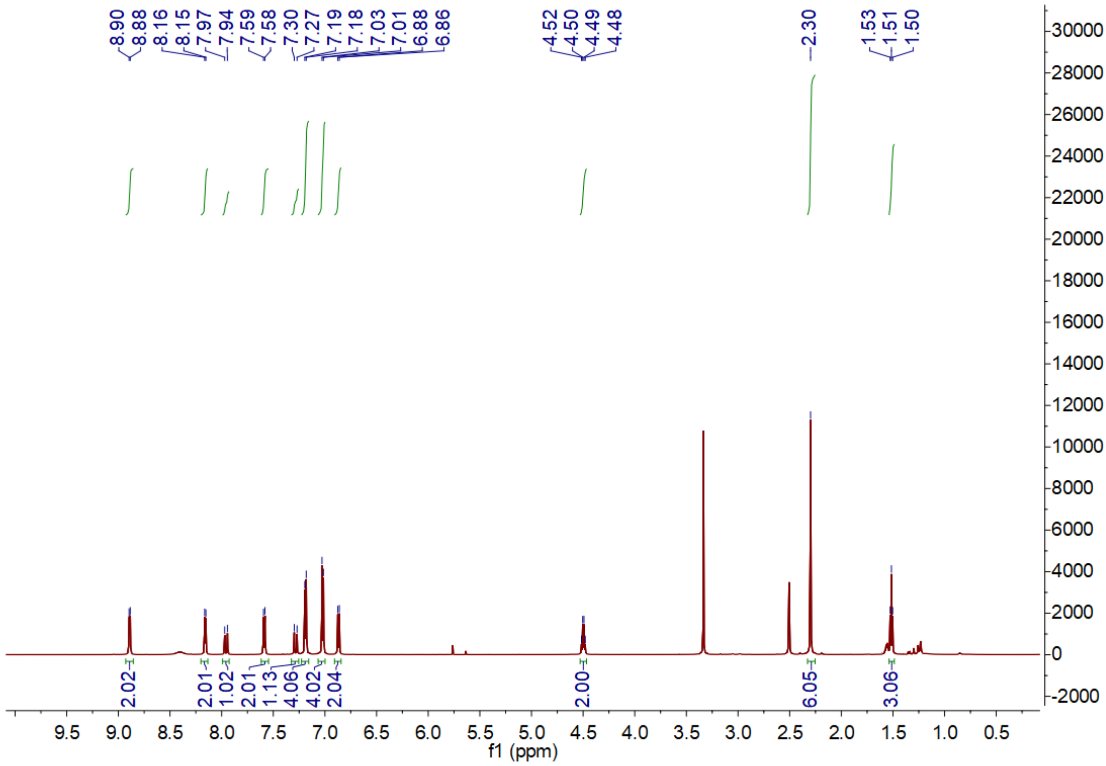
8.90
8.88
8.16
8.15
7.97
7.94
7.59
7.58
7.30
7.27
7.19
7.18
7.03
7.01
6.88
6.86
4.52
4.50
4.49
4.48
2.30
1.53
1.51
1.50
2.02
2.01
1.02
2.01
1.13
4.06
4.02
2.04
2.00
6.05
3.06
f1 (ppm)

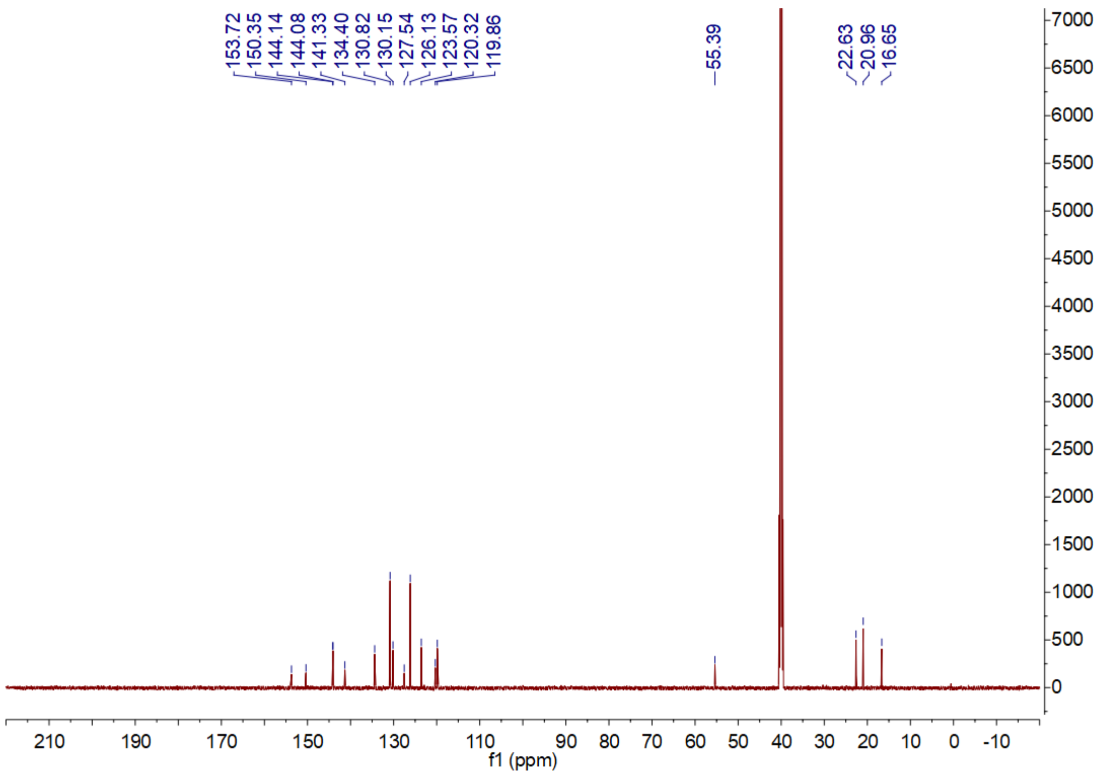
153.72
150.35
144.14
144.08
141.33
134.40
130.82
130.15
127.54
126.13
123.57
120.32
119.86
55.39
22.63
20.96
16.65
f1 (ppm)

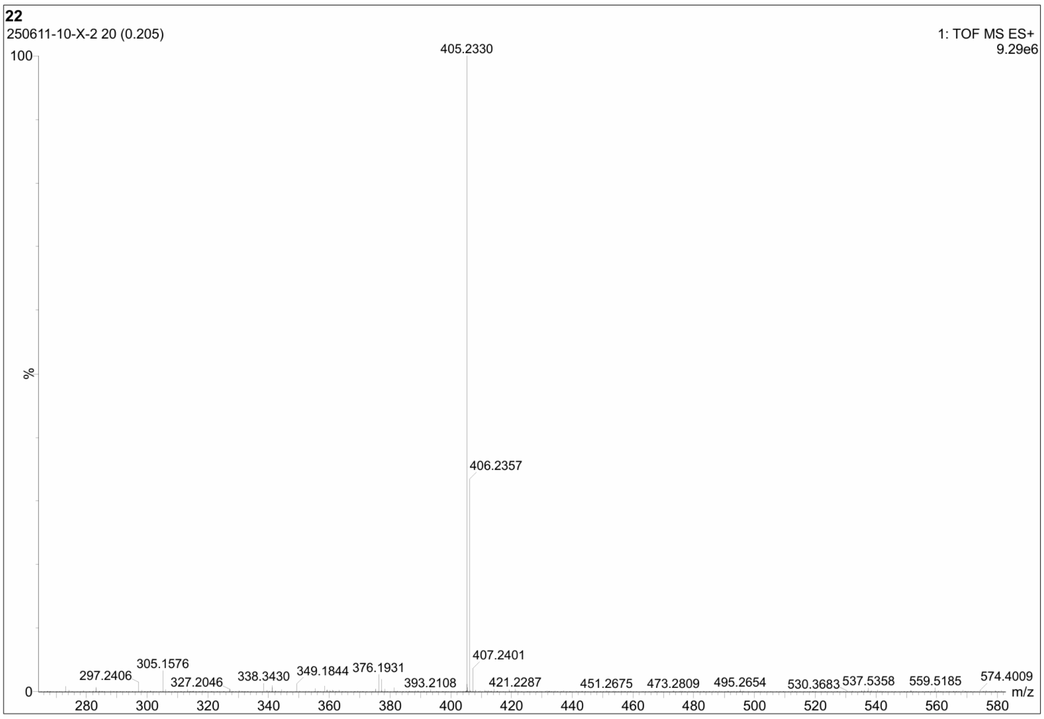

Figure S5. ^{1}H NMR (600 MHz, DMSO-d_6), ^{13}C NMR (151 MHz, DMSO-d_6) and HR-MS (ESI) (positive mode) spectra of SC-2.

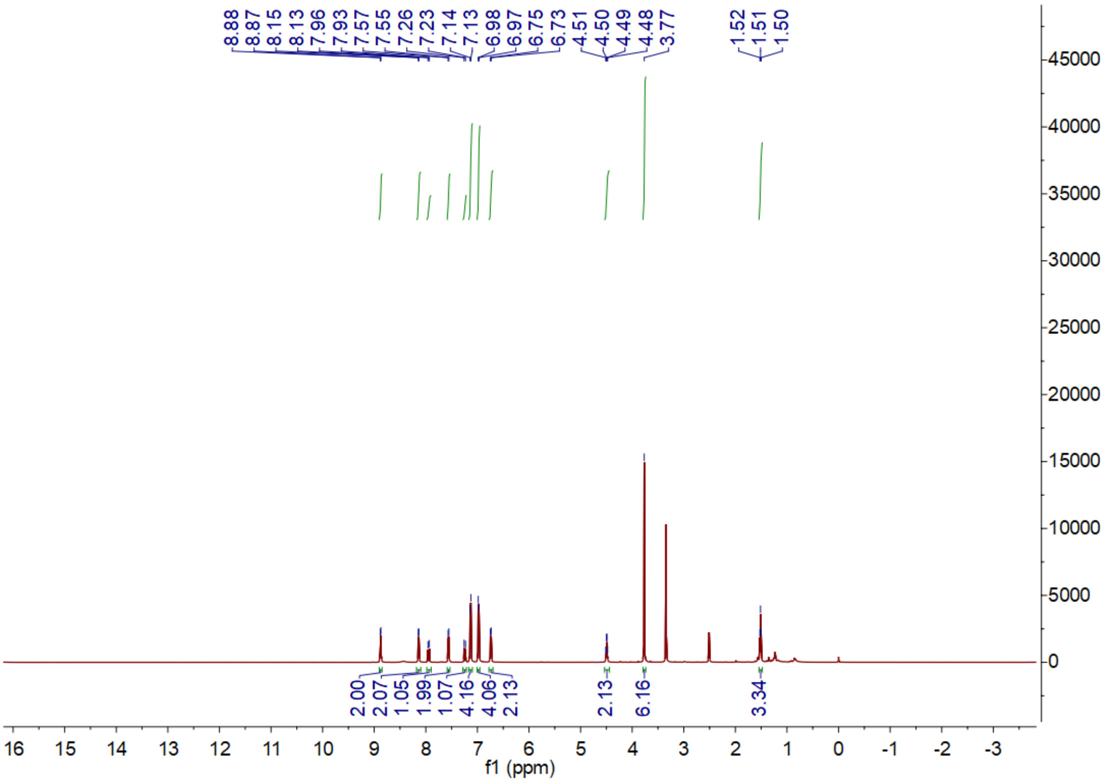

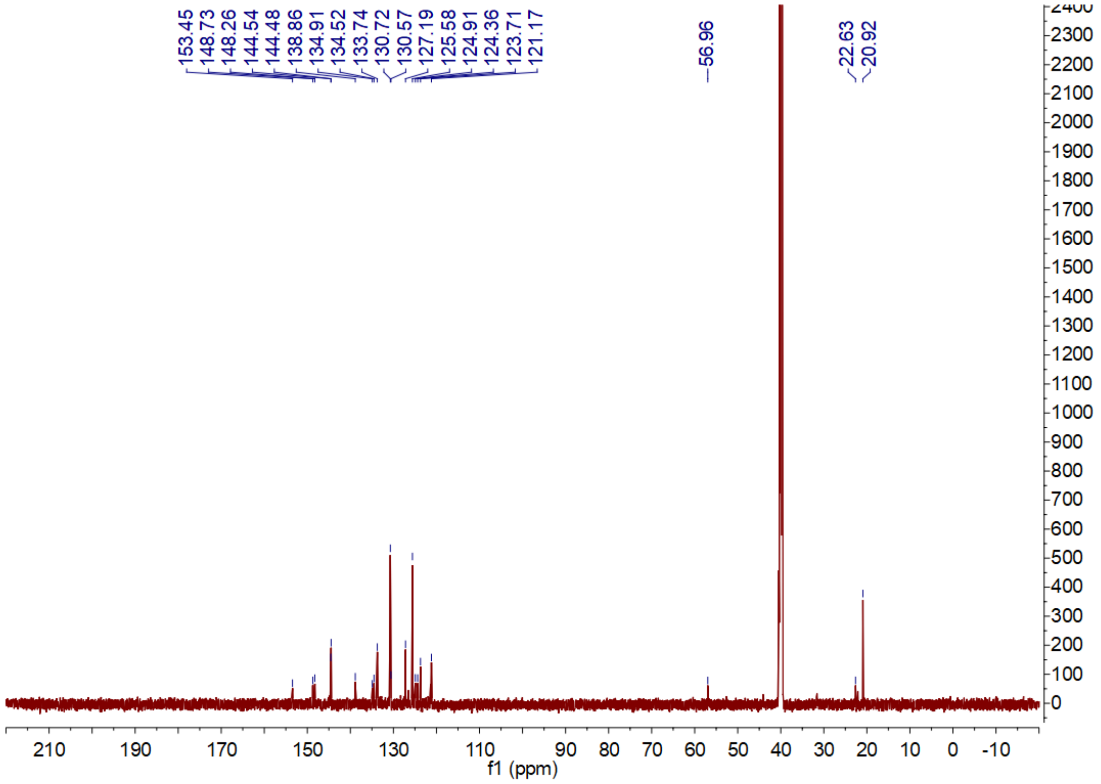

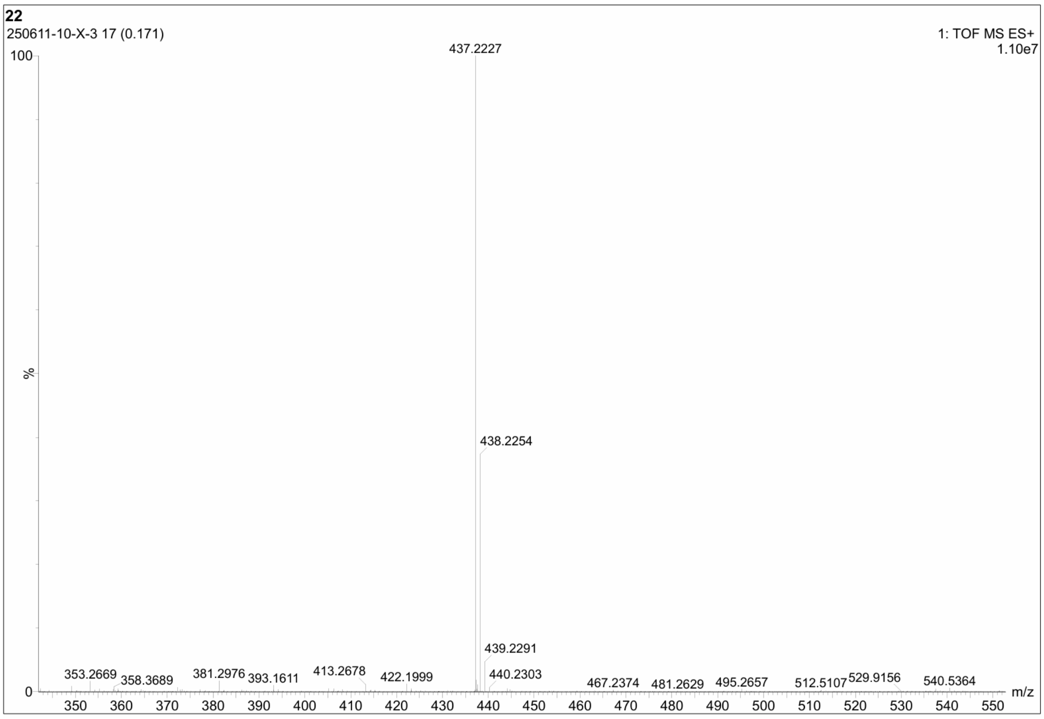

Figure S6. ^{1}H NMR (600 MHz, DMSO-d_6), ^{13}C NMR (151 MHz, DMSO-d_6) and HR-MS (ESI) (positive mode) spectra of SC-3.

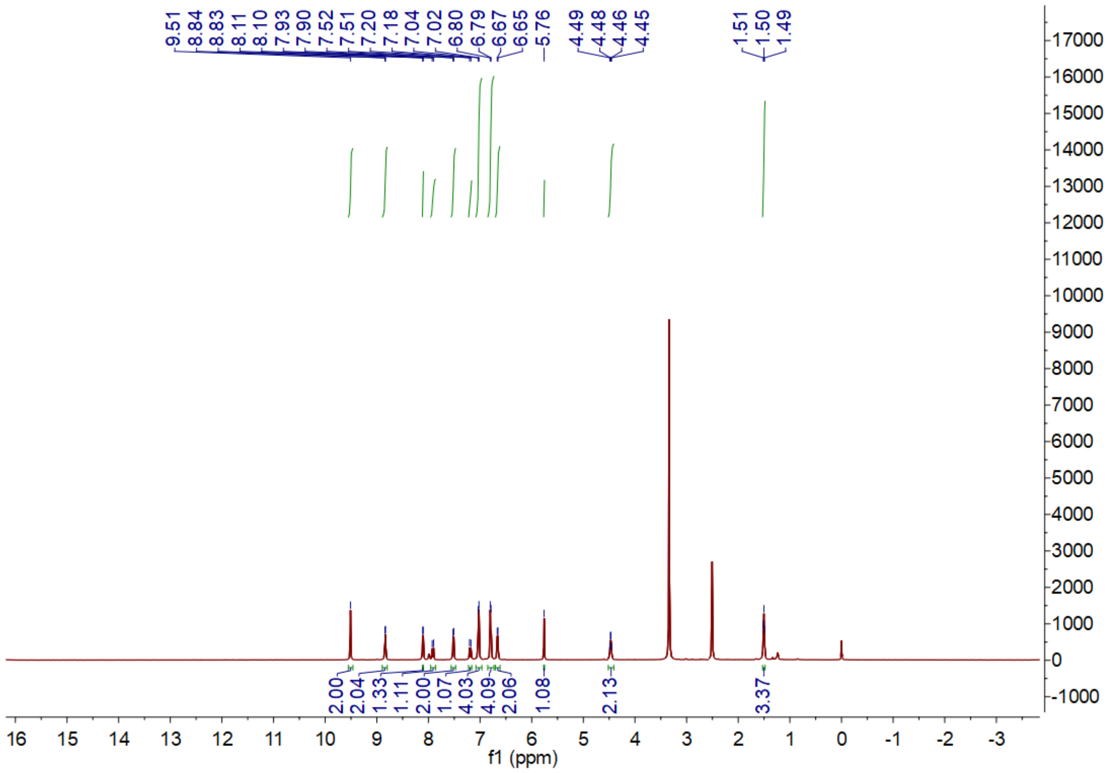
9.51
8.84
8.83
8.11
8.10
7.93
7.90
7.52
7.51
7.20
7.18
7.04
7.02
6.80
6.79
6.67
6.65
5.76
4.49
4.48
4.46
4.45
1.51
1.50
1.49
2.00
2.04
1.33
1.11
2.00
1.07
4.03
4.09
2.06
1.08
2.13
3.37
f1 (ppm)

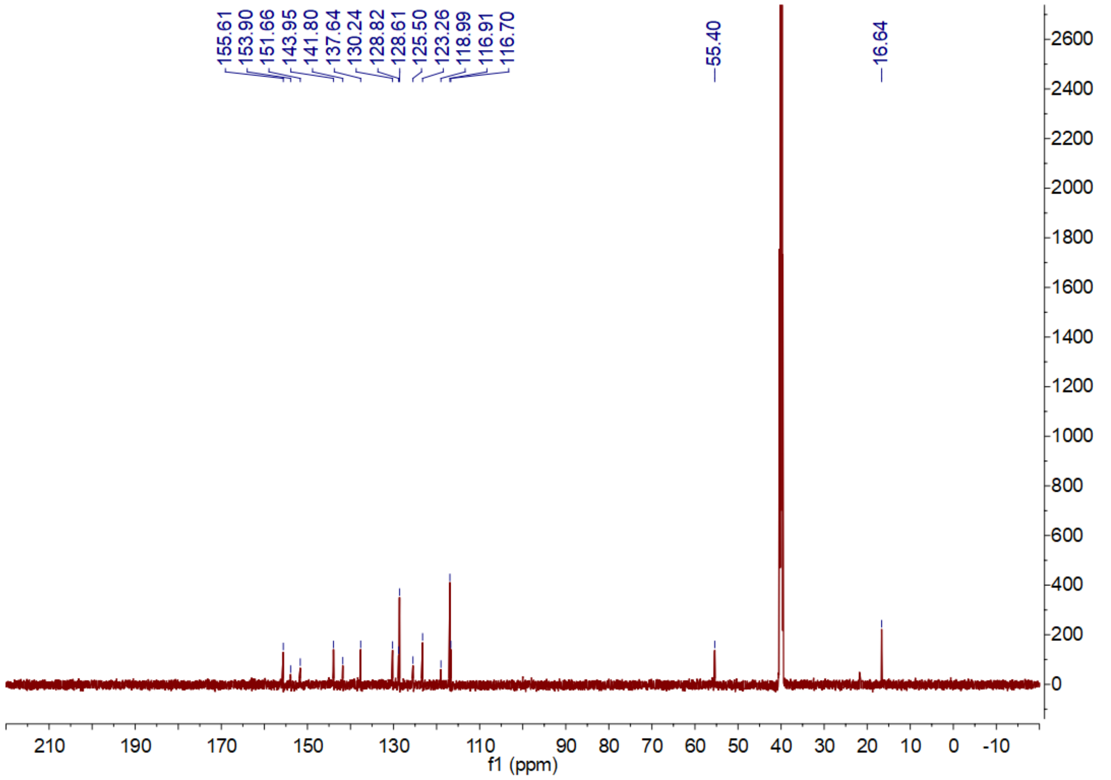
155.61
153.90
151.66
143.95
141.80
137.64
130.24
128.82
128.61
125.50
123.26
118.99
116.91
116.70
55.40
16.64
f1 (ppm)

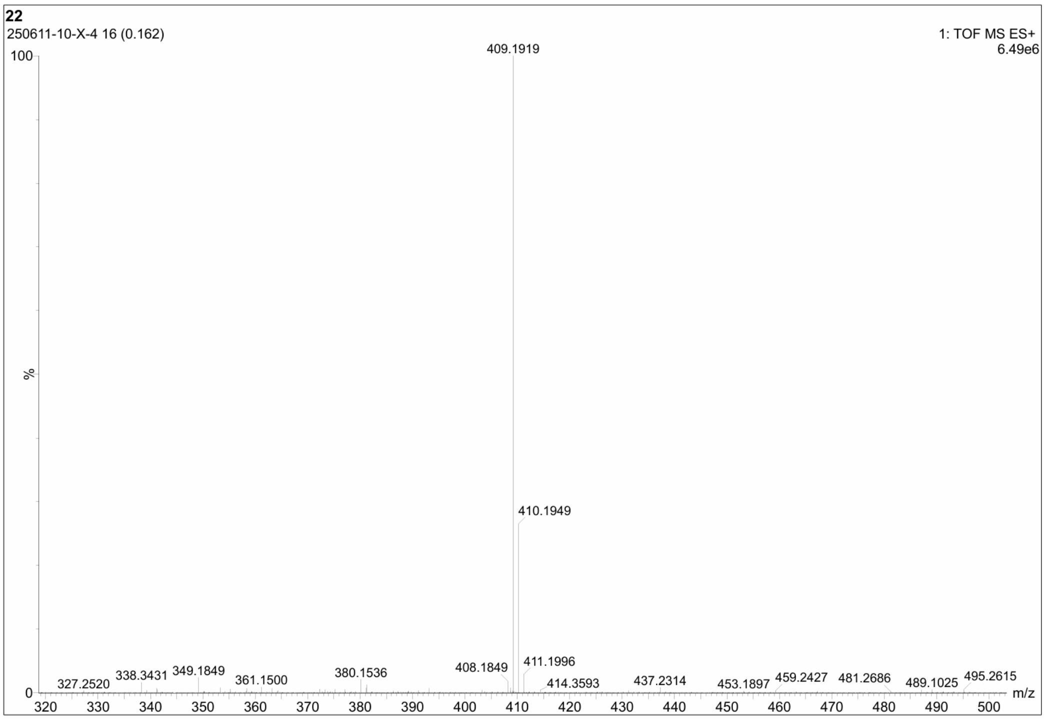

Figure S7. ^{1}H NMR (600 MHz, DMSO-d_6), ^{13}C NMR (151 MHz, DMSO-d_6) and HR-MS (ESI) (positive mode) spectra of SC-4.

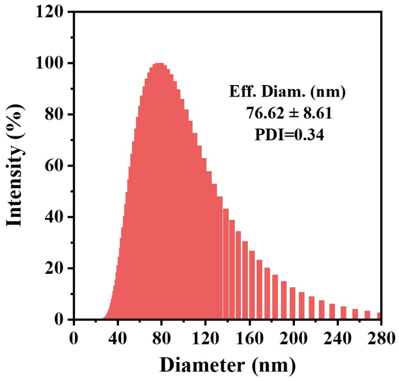

Figure S8. Particle size distribution of SC-3 in aqueous solution at concentration of 10 μM detected by dynamic light scattering (DLS).

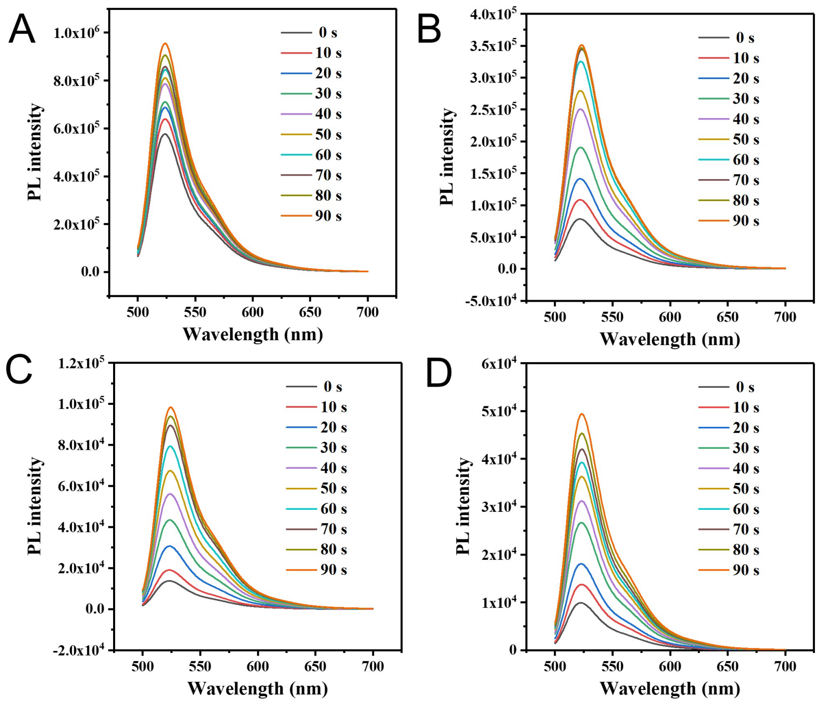

Figure S9. Fluorescence emission spectra of aqueous solutions containing (A) SC-1, (B) SC-2, (C) SC-3, and (D) SC-4 after addition of DCFH and irradiation for different times.

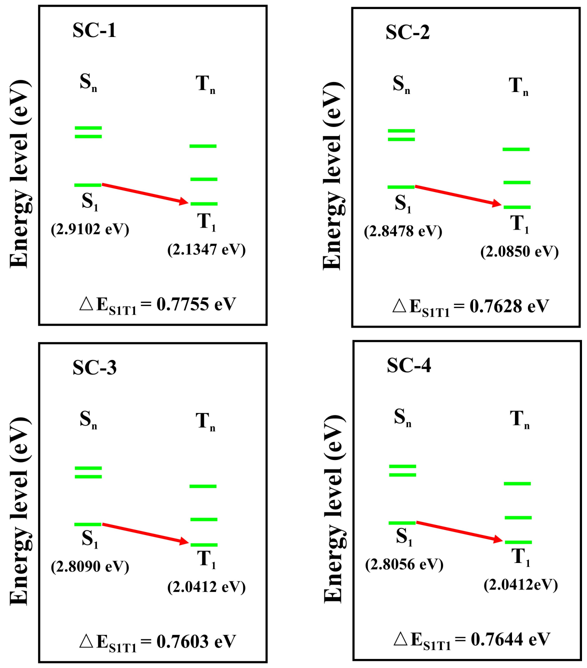

Figure S10. Singlet (S) and triplet (T) energy level distributions of SC-1, SC-2, SC-3, and SC-4 in water calculated by B3LYP/6-311G basis set.

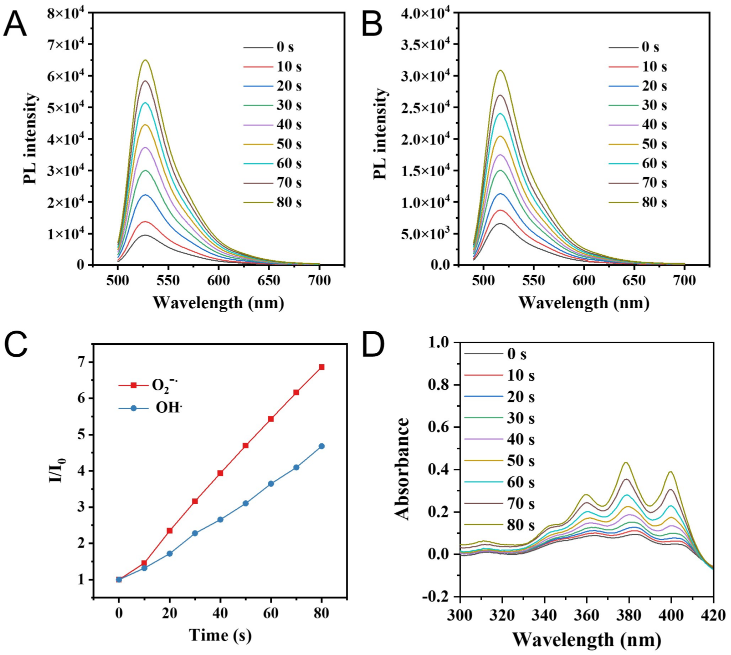

Figure S11. (A) Fluorescence emission spectra of aqueous solutions containing SC-3 after addition of DHR123 and irradiation for different durations. (B) Fluorescence emission spectra of aqueous solutions containing SC-3 after addition of HPF and irradiation for different durations. (C) Relative fluorescence intensity of SC-3-containing solutions after irradiation, using DHR123 and HPF as indicators. (D) Absorption spectra of aqueous solutions containing SC-3 after addition of 9,10-anthracenediyl-bis(methylene)dimalonic acid (ABDA) and irradiation for different durations, and the light source is white light with 40 mW/cm^2.

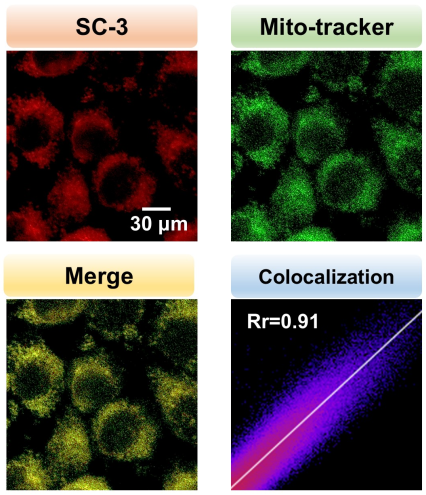

Figure S12. Mitochondrial co-localization images and quantitative co-localization

analysis of MDA-MB-231 cells after 4 h treatment with SC-3.

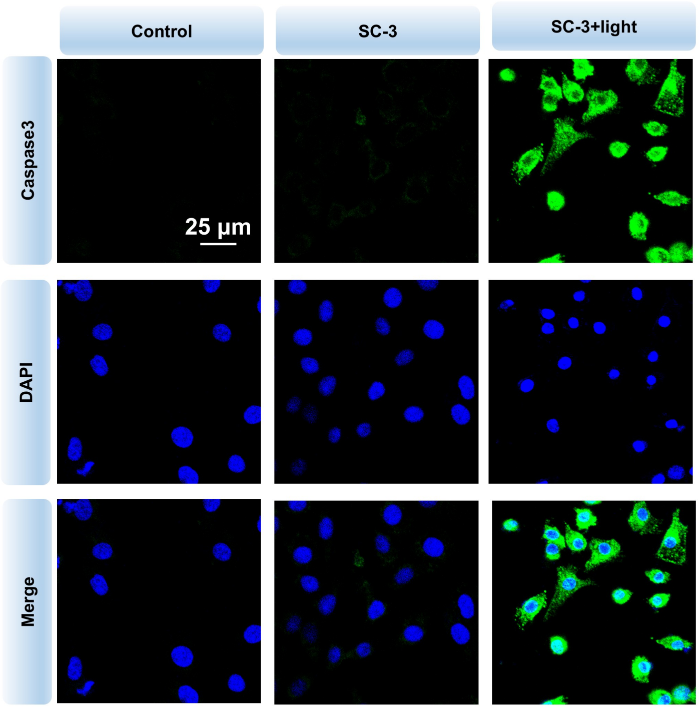

Figure S13. Immunofluorescence images of caspase3 in MDA-MB-231 cells after different treatment modalities. The light source is white light, the power is 40 mW/cm^2, and the irradiation time is 20 min.

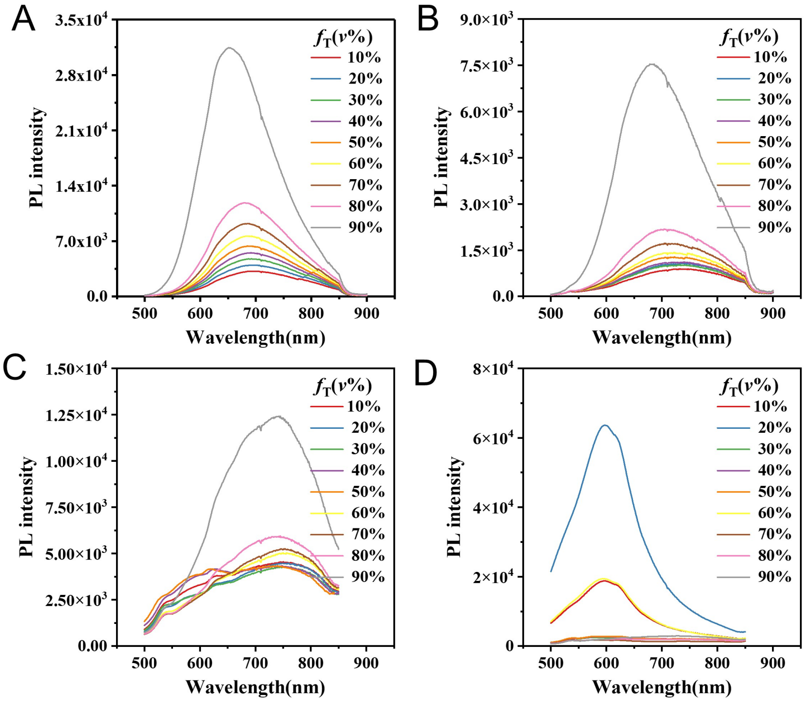

Figure S14. Fluorescence emission spectra of (A) SC-1, (B) SC-2, (C) SC-3, and (D)

SC-4 in toluene/dimethyl sulfoxide (Tol/DMSO) mixed solvents with different toluene ratios.

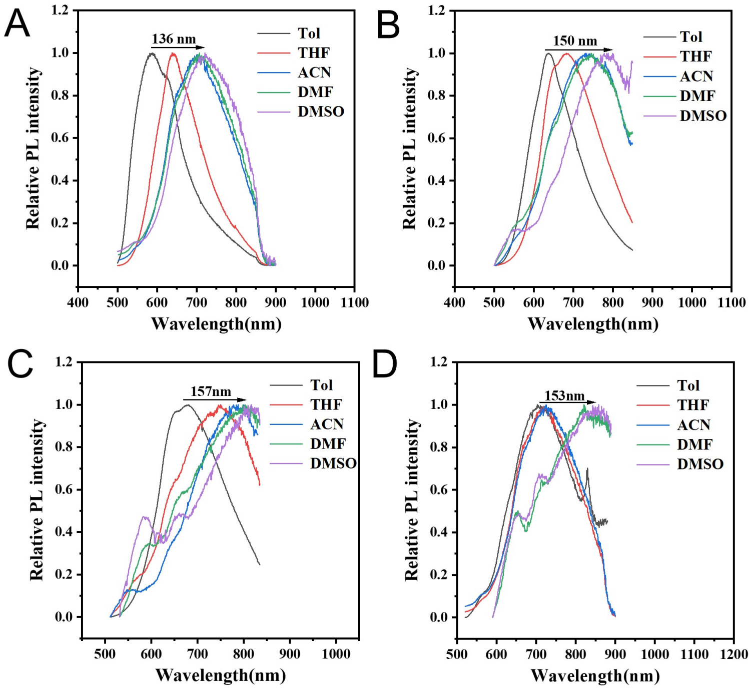

Figure S15. Fluorescence emission spectra of (A) SC-1, (B) SC-2, (C) SC-3, and (D) SC-4 in different solvents at a concentration of 10 μM, and THF is tetrahydrofuran.

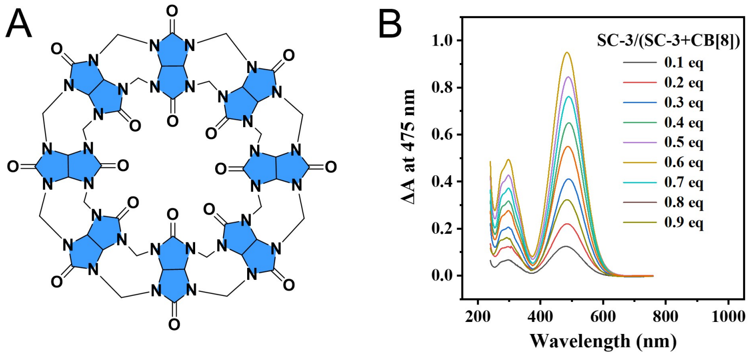

Figure S16. (A) The chemical structure of CB[8]. (B) Titration curves of SC-3 with CB[8].

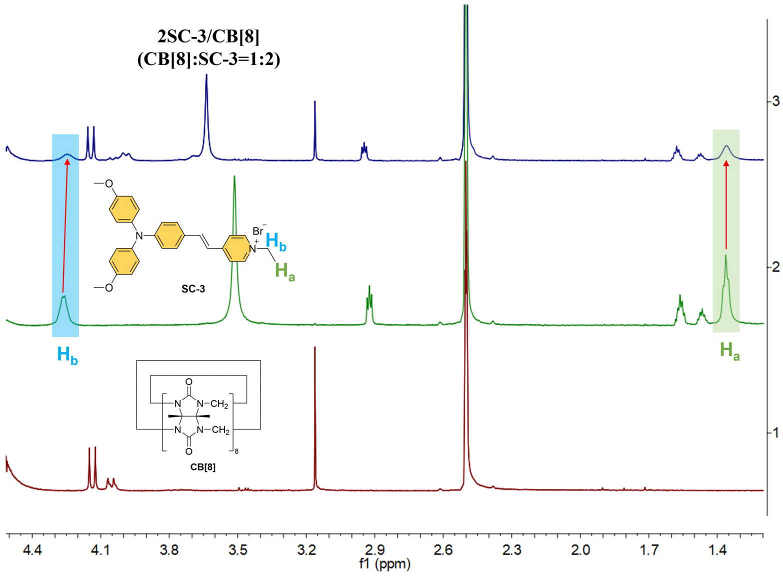

Figure S17. ^{1}H NMR (600 MHz) spectra of CB[8], SC-3, and 2SC-3/CB[8] in a mixed solvent of 10% DMSO-d_6 and 90% D_2O.

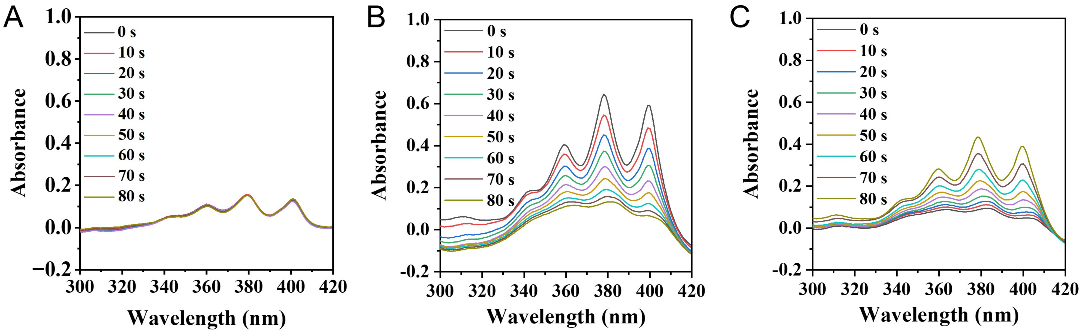

Figure S18. (A) Absorption spectra of aqueous solutions containing ABDA and irradiation for different durations. (B) Absorption spectra of aqueous solutions containing 2SC-3/CB[8] after addition of ABDA and irradiation for different durations. (C) Absorption spectra of aqueous solutions containing SC-3 after addition of ABDA and irradiation for different durations. The concentration of SC-3 is 10 µM, the concentration of 2SC-3/CB[8] is 5 µM, and the indicator is 10 µM. The light source is white light, the power is 40 mW/cm^2.

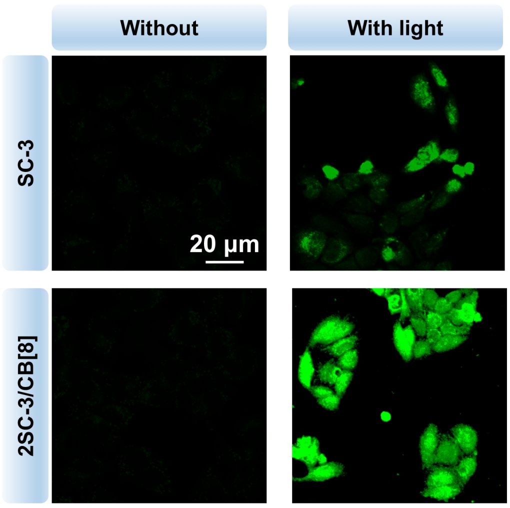

Figure S19. Fluorescence images of MDA-MB-231 cells incubated with SC-3 or 2SC-3/CB[8] before and after light irradiation, using SOSG as the $^1O_2$ indicator. The light source is white light, the power is 40 mW/cm^2, and the irradiation time is 5 min. The concentration of SC-3 is 10 μM, the concentration of 2SC-3/CB[8] is 5 μM.

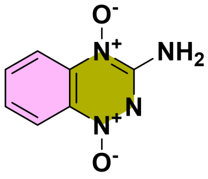

Tirapazamine

Figure S20. The chemical structure of Tirapazamine (TPZ).

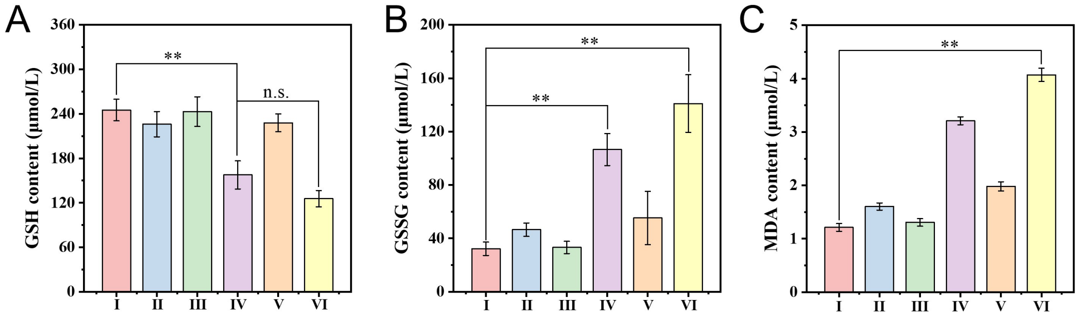

Figure S21. The contents of (A) GSH, (B) GSSG, and (C) MDA in MDA-MB-231 cells after different treatment modalities. I: Control, II: TPZ, III: 2SC-3/CB[8], IV: 2SC-3/CB[8]+light, V: 2SC-3/CB[8]+TPZ, VI: 2SC-3/CB[8]+TPZ+light, TPZ is tirapazamine, and the light source is white light, the power is 40 mW/cm^2, and the irradiation time is 20 min. Data were presented as mean ± SD (n = 3), and $^{**}P < 0.01$, n.s. represented no significance.

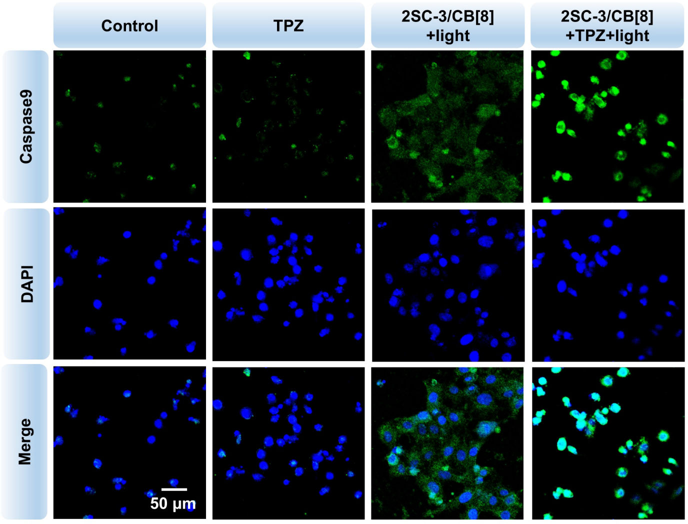

Figure S22. Immunofluorescence images of caspase9 in MDA-MB-231 cells after different treatment modalities. The light source is white light, the power is 40 mW/cm^2, and the irradiation time is 20 min. All concentrations of different compounds are 10 μM.

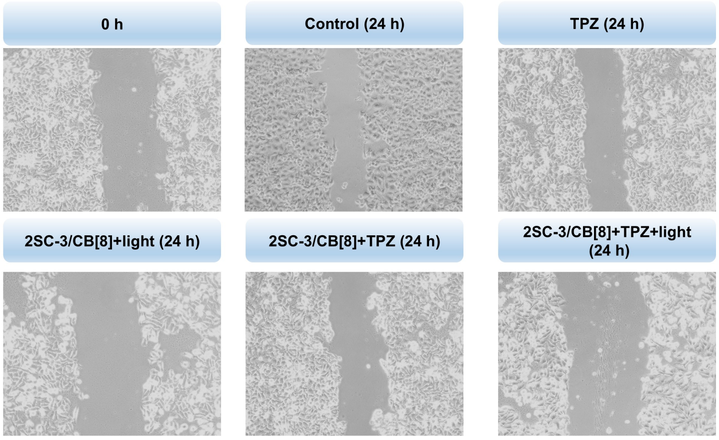

Figure S23. Tumor cell migration images of MDA-MB-231 cells after 24 h incubation following different treatment modalities. The light source is white light, the power is 40 mW/cm^2, and the irradiation time is 20 min. All concentrations of different compounds are 10 μM.

3. Tables about DFT calculation

Table S1. Calculated energy of the singlet (S) and triplet (T) excited states by B3LYP/6-311G basis set of SC-1, SC-2, SC-3 and SC-4 in water.

Compounds	SC-1	SC-2	SC-3	SC-4
**S_1 (eV)**	2.9102	2.8478	2.8090	2.8056
**S_2 (eV)**	4.1172	4.0636	3.9850	3.9839
**S_3 (eV)**	4.3306	4.2828	4.2068	4.2194
**S_4 (eV)**	4.4028	4.4304	4.4525	4.4743
**S_5 (eV)**	4.5680	4.5048	4.5077	4.5146
**S_6 (eV)**	4.6157	4.5504	4.5224	4.5375
**T_1 (eV)**	2.1347	2.0850	2.0487	2.0412
**T_2 (eV)**	3.3535	3.3187	3.2974	3.2989
**T_3 (eV)**	3.6433	3.6239	3.5499	3.5540
**T_4 (eV)**	3.8057	3.7538	3.7186	3.7329
**T_5 (eV)**	4.0260	3.9641	3.9113	3.9232
**T_6 (eV)**	4.1491	4.0864	4.0330	4.0106

4. Tables about cytotoxicity

Table S2. The cytotoxicity of different compounds co-incubated with MDA-MB-231 cells for 24 h was detected by 3-(4,5-dimethylthiazol-2-yl)-2,5-diphenyltetrazolium bromide (MTT), and the IC_{50} values of these compounds after co-treatment with white light were also calculated. White light irradiation for 20 min at 40 mW·cm^{-2}, and the incubator environment contained 21% O_2.

Compound	IC_{50} (μM)	
	**Dark**	**Light**
**TPZ**	95.62 ±10.21	—
**CB[8]**	> 100	—
**SC-3**	> 50	6.53 ± 0.674
**2SC-3/CB[8]**	> 50	3.25 ± 0.362
**2SC-3/CB[8]+TPZ**	> 50	3.04 ± 0.128

Table S3. The cytotoxicity of different compounds co-incubated with MDA-MB-231 cells for 24 h was detected by MTT, and the IC_{50} values of these compounds after co-treatment with white light were also calculated. White light irradiation for 20 min at 40 $mW·cm^{-2}$, and the incubator environment contained 8% O_2.

Compound	IC_{50} (μM)	
	Dark	Light
TPZ	36.21 ± 2.67	—
CB[8]	> 100	—
SC-3	> 50	8.51 ± 0.541
2SC-3/CB[8]	> 50	4.95 ± 0.562
2SC-3/CB[8]+TPZ	28.24 ± 1.95	0.95 ± 0.047

Table S4. The cytotoxicity of different compounds co-incubated with MDA-MB-231 cells for 24 h was detected by MTT, and the IC_{50} values of these compounds after co-treatment with white light were also calculated. White light irradiation for 20 min at 40 $mW·cm^{-2}$, and the incubator environment contained 2% O_2.

Compound	IC_{50} (μM)	
	Dark	Light
TPZ	22.43 ± 3.86	—
CB[8]	> 100	—
SC-3	> 50	9.04 ± 0.364
2SC-3/CB[8]	> 50	6.23 ± 0.754
2SC-3/CB[8]+TPZ	18.56 ± 2.67	0.42 ± 0.064

Table S5. The cytotoxicity of different compounds co-incubated with human umbilical vein endothelial cells (HUVECs) for 24 h was detected by MTT.

**Compound**	**IC_{50} (μM)**
**TPZ**	> 100
**CB[8]**	> 100
**SC-3**	> 50
**2SC-3/CB[8]**	> 50
**2SC-3/CB[8]+TPZ**	> 50
